# Distinguishing self from non-self RNA by editing-specific inosine patterns

**DOI:** 10.1093/nar/gkag845

**Published:** 2026-08-24

**Authors:** Rajagopal Varada, Alina F Leuchtenberger, Cornelia Vesely, Beata Kaczmarek, Hamid Mansouri Khosravi, Therese C Mandl, Katarina Milanovic, Kasra Honarmand Tamizkar, Vinod Rajendra, Hannes Senoner, Linda Steinbichl, Marija Borojevic, Andy Sombke, Katy Schmidt, Margret Eckhard, Ivo L Hofacker, Carl Walkley, Jacki E Heraud-Farlow, Ernesto Picardi, Carrie Bernecky, Michael F Jantsch

**Affiliations:** Center of Anatomy and Cell Biology, Division of Cell & Developmental Biology, Medical University of Vienna, Schwarzspanierstrasse 17, Vienna A-1090, Austria; Center for Integrative Bioinformatics Vienna (CIBIV) Max Perutz Labs Vienna Biocenter Campus (VBC), Dr. Bohr Gasse 9, Vienna A-1030, Austria; Center of Anatomy and Cell Biology, Division of Cell & Developmental Biology, Medical University of Vienna, Schwarzspanierstrasse 17, Vienna A-1090, Austria; Institute of Science and Technology Austria (ISTA), Am Campus 1, 3400 Klosterneuburg, Austria; Center of Anatomy and Cell Biology, Division of Cell & Developmental Biology, Medical University of Vienna, Schwarzspanierstrasse 17, Vienna A-1090, Austria; Center of Anatomy and Cell Biology, Division of Cell & Developmental Biology, Medical University of Vienna, Schwarzspanierstrasse 17, Vienna A-1090, Austria; Center of Anatomy and Cell Biology, Division of Cell & Developmental Biology, Medical University of Vienna, Schwarzspanierstrasse 17, Vienna A-1090, Austria; Center of Anatomy and Cell Biology, Division of Cell & Developmental Biology, Medical University of Vienna, Schwarzspanierstrasse 17, Vienna A-1090, Austria; Center of Anatomy and Cell Biology, Division of Cell & Developmental Biology, Medical University of Vienna, Schwarzspanierstrasse 17, Vienna A-1090, Austria; Center of Anatomy and Cell Biology, Division of Cell & Developmental Biology, Medical University of Vienna, Schwarzspanierstrasse 17, Vienna A-1090, Austria; Center of Anatomy and Cell Biology, Division of Cell & Developmental Biology, Medical University of Vienna, Schwarzspanierstrasse 17, Vienna A-1090, Austria; Center of Anatomy and Cell Biology, Division of Cell & Developmental Biology, Medical University of Vienna, Schwarzspanierstrasse 17, Vienna A-1090, Austria; Center of Anatomy and Cell Biology, Division of Cell & Developmental Biology, Medical University of Vienna, Schwarzspanierstrasse 17, Vienna A-1090, Austria; Center of Anatomy and Cell Biology, Division of Cell & Developmental Biology, Medical University of Vienna, Schwarzspanierstrasse 17, Vienna A-1090, Austria; Center of Anatomy and Cell Biology, Division of Cell & Developmental Biology, Medical University of Vienna, Schwarzspanierstrasse 17, Vienna A-1090, Austria; Department of Theoretical Chemistry, University of Vienna, Währinger Strasse 17, 1090 Vienna, Austria; Research Group Bioinformatics and Computational Biology, Faculty of Computer Science, University of Vienna, Vienna 1090, Austria; Hudson Institute of Medical Research and Department of Molecular and Translational Science, Monash University, 27–31 Wright Street, Clayton, VIC 3168, Australia; Hudson Institute of Medical Research and Department of Molecular and Translational Science, Monash University, 27–31 Wright Street, Clayton, VIC 3168, Australia; Department of Bioscience, Biotechnology and Biopharmaceutics, University of Bari Aldo Moro, University Campus “Ernesto Quagliariello”, Via Orabona 4, Bari, Italy; Institute of Science and Technology Austria (ISTA), Am Campus 1, 3400 Klosterneuburg, Austria; Center of Anatomy and Cell Biology, Division of Cell & Developmental Biology, Medical University of Vienna, Schwarzspanierstrasse 17, Vienna A-1090, Austria

## Abstract

The cytoplasmic antiviral sensor MDA5 is activated by double-stranded RNAs. Endogenous double-stranded RNAs are modified by the A-to-I RNA-editing ADAR family to prevent activation of MDA5. *In vivo*, cytoplasmic ADAR1p150 is critically required to suppress MDA5 activation, yet the editing signature of all ADAR isoforms is strongly overlapping in mice. Further, it is not clear how A-to-I modifications in dsRNA prevent MDA5 activation. Here we show that 3′ UTRs harboring inverted repeats activate MDA5 in vitro and in cells. *In vitro* editing by either ADAR isoform leads to editing at overlapping hotspot regions and prevents MDA5 activation *in vitro* and in cells. Remarkably, only inosines introduced by RNA editing are capable of suppressing MDA5 activation, while replacing guanosines with inosines during *in vitro* transcription has no impact on MDA5 activation. A comparison of inosines introduced by ADAR1p150 *in vitro*, in cells, and *in vivo* suggests that a small number of A-to-I conversions may be critically required to suppress MDA5 activation. As those critical editing events are predominantly altering A:U basepairs into I:U wobble basepairs, we suggest that the helical distortion introduced by those wobble pairs may prevent MDA5 polymerization and thus downstream activation of the type I interferon response.

## Graphical Abstract

**Graphical Abstract:**
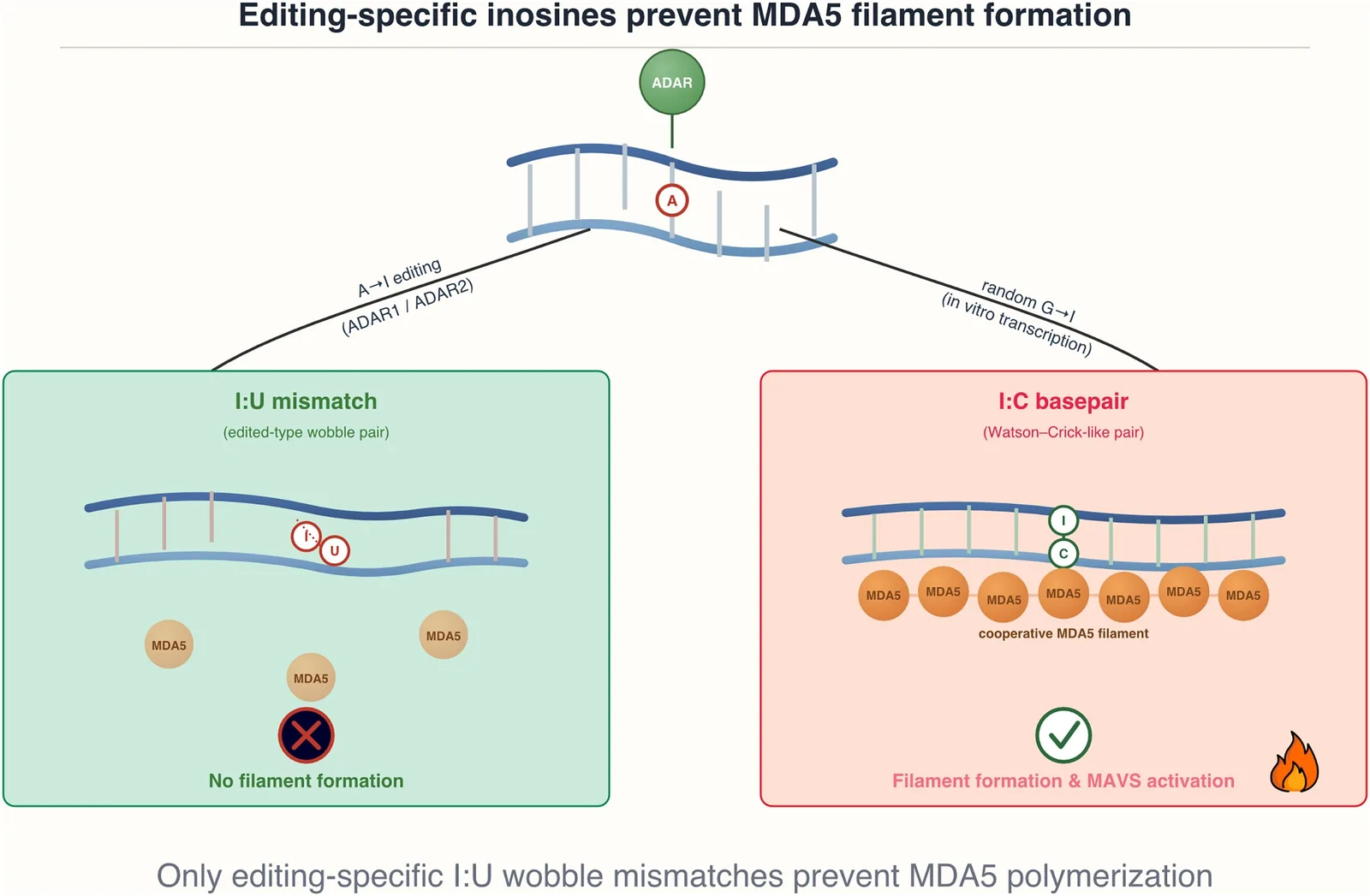

## Introduction

Viral infection can lead to the accumulation of cytoplasmic double-stranded RNAs (dsRNAs). In mammalian host cells, these RNAs are typically recognized by cellular dsRNA-binding proteins such as melanoma differentiation antigen 5 (MDA5) and protein kinase RNA-activated (PKR) [1, 2]. When MDA5 binds dsRNAs, it polymerizes and hydrolyses ATP while testing the dsRNA quality. Aggregation of the CARD domains of MDA5 leads to downstream activation of MAVS [3, 4]. This, in turn, leads to activation of antiviral responses and interferon production. PKR activation, in contrast, leads to translational shutdown [5, 6]. Interestingly, dsRNAs are also abundantly formed by base pairing of repetitive elements that are located in inverted orientation within endogenous transcripts [7]. In mammals, repetitive elements of the short interspersed element (SINE) family are most abundant and therefore frequently form intramolecular double-stranded regions [8]. Endogenous dsRNAs are normally masked as “self” by A-to-I RNA editing through adenosine deaminases acting on RNA (ADARs), which prevents inadvertent activation of antiviral signaling [9–11]. In mammals, ADAR1 (*Adar*) and ADAR2 (*Adarb1*) can edit dsRNAs. An interferon-inducible ADAR1p150 isoform is mainly localized to the cytoplasm and is critically required to suppress antiviral signaling, while a constitutively expressed ADAR1p110 isoform also edits endogenous dsRNAs but is not sufficient to suppress antiviral signaling [12, 13]. Indeed, the extended amino terminus of ADAR1p150 carries a Z-DNA binding domain that also regulates antiviral responses by sensing RNAs in Z-conformation [14]. Further, ADAR1p150 has also been shown to suppress activation of PKR [15, 16]. Consistently, ADAR1 depletion leads to the accumulation of dsRNAs, subsequent stress granule formation, and activation of dsRNA-binding proteins such as MDA5 or PKR and others to dsRNAs [17–19]. ADAR2 is also localized to the nucleus but its expression is restricted to a few tissues, including nervous tissue and the gastrointestinal tract.

In humans, reduced ADAR1 activity leads to Aicardi–Goutières syndrome [20–22], and it is generally believed that the underlying cause is the inadvertent activation of the antiviral sensor MDA5. This notion is corroborated by the situation in mice where the embryonic lethality of mice with a catalytically inactive ADAR1 can be fully rescued by a concomitant MDA5 deletion [10].

While the editing substrates of ADAR1 and ADAR2 seem distinct in humans, a large overlap in editing sites is seen in mice [23, 24]. Still, neither ADAR2 nor ADAR1p110 can genetically substitute for loss of ADAR1p150. Loss of ADAR1p150 triggers the innate immune system in mice, leading to embryonic lethality [25, 26]. The spectrum of human disease mutations suggests the same to be true in humans. Thus, ADAR1p150 can suppress immune responses via three different pathways. The ZBD in ADAR1p150 seemingly competes for RNAs in Z-conformation. Mutations in the ZBD lead to an activation of Z-RNA binding protein (ZBP), subsequently inducing necroptosis [27]. The dsRBDs of ADAR1 seemingly compete with PKR for the binding of dsRNA but may also physically interact with PKR to prevent PKR activation [15, 16]. Lastly, the catalytic activity of ADAR1 is essential to prevent activation of MDA5 [10, 14, 28]. Despite the large overlap in editing patterns between ADAR1p150, ADAR1p110, and ADAR2 observed in mice, only ADAR1p150 seems critically required to antagonize MDA5 and PKR activation [12]. ADAR1p110 deficiency leads to a high postnatal lethality but lack of ADAR1p110 activity does not lead to MDA5 activation [29]. This finding is quite surprising given that both ADAR1p110 and ADAR1p150 show a significant overlap in the targeted sites.

Given that inhibition of ADAR1p150 can activate the innate immune system and can help to overcome PD1 checkpoint blockade, an important suppressor of cancer immune therapies, it is important to identify endogenous RNAs that can activate MDA5 in the absence of ADAR1 [30]. Likewise, it is necessary to understand how editing of endogenous dsRNAs can prevent MDA5 activation. To address these points, we have characterized endogenous dsRNAs in the mouse that can activate MDA5 when not edited. Importantly, using *in vivo* and *in vitro* editing assays, we decipher editing sites critically required to suppress an innate immune response. We can show that selective introduction of a few inosines by RNA editing at a few critical sites is sufficient to repress innate immune activation and stress signaling. In contrast, stochastic substitution of guanosines by inosines during *in vitro* transcription in ds RNAs fails to suppress MDA5 activation and stress signaling. This indicates that I:U wobble basepairs may suppress MDA5 activation while I:C basepairs fail to do so.

## Materials and methods

### Ethics statement

Mouse breeding of genetically modified strains was performed in the mouse facility of the Medical University of Vienna. Mouse breeding and handling were approved by the in-house animal committee and the Federal Ministry for Science and Education, permit numbers BMWFW-66.009/0267-WF/V/3b/2017 and BMWFW-66.009/0268-WF/V/3b/2017. Mice were genotyped on phalanx biopsies. For organ removal, animals were euthanized within 12 days postpartum by cervical dislocation.

### Mice

Adar-deficient mice were obtained from the Seeburg lab [25]. MAVS-deficient mice were obtained from the Jackson Laboratories [6]. Double mutant mice were generated by intercrossing these strains followed by backcrossing to Bl6^J^. Genotyping was performed by PCR on finger biopsies.

### Total RNA isolation from mouse liver

Liver tissues from 8–9 days old *Mavs^−/−^* and *Adar1^−/−^/Mavs^−/−^* mutant mice were extracted and weighed, with 100 mg used for total RNA isolation. Briefly, 1 ml of TRIzol and 1.4 mm zirconium oxide beads were added to 100 mg of liver tissue in 2 ml screw-cap tubes. The tissues were homogenized using a tissue homogenizer (MP Biomedicals). Following homogenization, 200 μl of chloroform was added and mixed well. Samples were centrifuged at 4°C, 15 000 rpm for 15 min to separate into layers. The aqueous layer was collected, and an equal volume of PCI (phenol:chloroform:isoamyl alcohol) was added, followed by another centrifugation at 4°C, 15 000 rpm for 10 min. The aqueous layer was again collected, and an equal volume of chloroform was added, followed by centrifugation at 4°C, 15 000 rpm for 10 min. To the final aqueous layer, 2× the volume of 96% ethanol and 1/10 volume of 3M sodium acetate (pH 5.3) were added. The tubes were kept at −20°C for a minimum of 2 h or overnight for RNA precipitation. Afterwards, the samples were centrifuged at 4°C, 15 000 rpm for 15 min. The supernatant was discarded, and 1 ml of 70% ethanol was added to the tubes, which were centrifuged again under the same conditions. The supernatant was discarded, and the pellets were dried at 37°C for ~5 min. Finally, the pellets were resuspended in the desired volume of nuclease-free DEPC-treated ddH_2_O.

The RNA samples were treated with DNase I (NEB) to remove genomic DNA contamination. For each sample, 10 μl of 10× DNase I Reaction Buffer, 15 μl DNase I (DNase-free), 1 μl RNase Inhibitor, and 74 μl nuclease-free ddH_2_O were added. The mixture was incubated at 37°C for 2 h. After DNase I treatment, samples were extracted with 2× PCI, 1× chloroform, and 96% ethanol. RNA was quantified by Qubit fluorimetry (Thermo Fisher, Waltham, MA).

### Isolation of RNA from Adar1p150^-/-^ mice

Immortalized mouse embryonic fibroblasts (MEFs) were generated from *Adar1p150^−/−^* mice as previously described [12]. The cells were plated in 10 cm dishes and treated for 24 h with 250 U/ml recombinant murine interferon beta (PBL Assay Science; PBL-12405) or vehicle. RNA was extracted using the Nucleospin RNA plus RNA extraction kit (Machery Nagel) according to the manufacturer’s instructions. The RNA was then dried in Novogene RNA ambient tubes for international shipping at room temperature.

### Purification of recombinant MDA5 protein

Murine MDA5 cDNA was cloned into a pET-24a-d(+) vector with a His-tag and expressed in BL21 (DE3) Rosetta *E. coli*. Expression was induced overnight at 20°C with 0.1 mM IPTG. Cells were suspended in a buffer containing 20 mM Tris–HCl (pH 8.0), 500 mM NaCl, 2 mM DTT, 10% glycerol, 1 mM PMSF, 0.1% Triton X-100, and 10 mM imidazole. Cells were lysed with 1 mg/ml lysozyme for 30 min at 25°C, followed by 10 µg/ml DNase I treatment for 30 min at 25°C, and sonication. The lysate was centrifuged at 30 000 rpm for 20 min at 4°C and filtered through 0.45 µm sterile filters before loading onto a Ni-NTA column. The recombinant protein was purified by Ni-NTA chromatography using an imidazole gradient and then subjected to size exclusion chromatography in a buffer containing 20 mM HEPES (pH 7.5), 150 mM NaCl, and 2 mM DTT. The purified protein was stored in a buffer with 20 mM HEPES (pH 7.5), 150 mM NaCl, 2 mM DTT, and 30% glycerol. The quality of the recombinant protein was verified by sodium dodecyl sulfate–polyacrylamide gel electrophoresis (SDS–PAGE).

### Gel electrophoresis

To check the quality of RNAs, 1 µg of RNA was mixed with 2× RNA loading dye (Thermo Scientific) and heated at 70°C for 3 min. The samples were then loaded onto an 8M Urea PAGE (6% acrylamide) gel alongside the RNA RiboRuler High Range RNA Ladder (Thermo Scientific). After electrophoresis, the gel was stained with EtBr.

### RNase protection assay and RNA library preparation

Total RNA isolated from the liver tissue of ADAR1 WT and ADAR1-deficient mice was kept at 70°C for 5 min and allowed to refold in a buffer containing 20 mM HEPES (pH 7.5), 50 mM NaCl, 2 mM MgCl₂ at room temperature for 15 min. The RNA was then incubated with recombinant purified MDA5Δ2CARD protein in a buffer containing 20 mM HEPES (pH 7.5), 50 mM NaCl, 2 mM MgCl₂, and 2 mM DTT. Next, 2 μg of total RNA from WT and ADAR1-deficient mice was incubated with increasing amounts of recombinant MDA5Δ2CARD protein (100–400 μM) in the presence of 1 mM ATP at 25°C for 10 min. The samples were then treated with a 1:100 dilution of RNase A/T1 mix (Thermo Scientific) for 5 min to digest unbound RNAs. Untreated samples and samples treated with only RNA and RNase served as controls. RNase A/T1 digestion was quenched by adding five volumes of TRIzol reagent, and RNA was isolated using the 2× PCI, 1× CI, and 96% ethanol precipitation method. The samples were then loaded onto an 8M Urea PAGE (6% acrylamide) gel to check for protected RNA species. Protected RNA was purified using the RNAeasy Mini Kit (QIAGEN) to remove small digestion products. rRNA was depleted with the NEBNext rRNA Depletion Kit (E6310, E6350). Using the manufacturer’s protocol, cDNA libraries were prepared with the NEBNext Ultra II Directional RNA Library Prep Kit for Illumina (NEB E7760, E7765).

### RNA-seq data analysis

The cDNA libraries were quality-controlled using a Bioanalyzer at the Medical University of Vienna Core Sequencing Facility. Libraries from control samples (no MDA5 and no RNase) and MDA5-protected samples from both WT and ADAR1 mutant groups were diluted to 0.5 nmol. For the final library pool, 2 µl of each library was used. Sequencing was performed on an Illumina NextSeq instrument with single-ended 75 bp reads. Both the sequencing reactions and subsequent quality control were conducted at the Core Sequencing Facility. Each library, from control and MDA5-protected samples, yielded ~4–12 million reads. Raw sequence data were pre-processed using Trim Galore (version 0.6.6) [31] to trim Illumina adapters, remove low-quality ends (quality score below 20), and discard reads shorter than 20 bp. The processed reads were aligned to the mouse genome (GRCm38) using NextGenMap version 0.5.5 [32]. Further quality control assessments were performed using Qualimap version 2.2.2 [33]. Sample comparisons, including multi-BAM summary statistics, correlation plots, and PCA plots, were performed using Deeptools version 3.5.0 [34].

### Peak calling

Peak calling was performed using the Genrich tool on aligned regions. Reads were normalized to the input sequencing of the corresponding genetic backgrounds. For strand-specific peak detection, each chromosome’s strands were treated as separate chromosomes; this adaptation was implemented using samtools version 1.7 [35]. Genrich version 0.6.1 [36] was used to call peaks for the “no MDA5, but RNase” and “MDA5 & RNase” libraries, using the corresponding “no MDA5 & no RNase” libraries as controls. Additionally, peaks were called for the “no MDA5 & no RNase” libraries. The peak calling parameters included a maximum adjusted *P*-value of 0.01, a minimum area under the curve (AUC) of 500, and a maximum gap of 20 bp between significant sites. Blacklisted regions from ENCODE (mm10_blacklist.bed, available at ENCODE Blacklist [37]) and rRNA entries from RepeatMasker [38] were excluded from the analysis. Quality checks were conducted using the Integrative Genomics Viewer version 2.8.6 [39].

### Peak calling pipeline

The peak pipeline involved several steps. First, RNase replicates were joined by retaining only those peaks that were within 200 bp of at least one peak in another replicate. Next, the resulting RNase peaks were subtracted from each MDA5 sample on a base pair (bp)-wise basis, where only the overlapping region between an MDA5 peak and an RNase peak was removed, not the entire peak. Any resulting peak shorter than 10 bp was then removed. Subsequently, the MDA5 replicates were joined by retaining only those peaks that were within 200 bp of a peak in another replicate.

Subsequently, three peak lists were formed: one exclusively listing MDA5 peaks from the *Adar^−/−^; Mavs^−/−^* (where *Mavs^−/−^* peaks and *Mavs^−/−^* RNase peaks were subtracted from the *Adar^−/−^; Mavs^−/−^* peaks on a bp-wise basis), one exclusively listing *Mavs^−/−^* peaks (where *Adar^−/−^; Mavs^−/−^* peaks and *Adar^−/−^; Mavs^−/−^* RNase peaks were subtracted from the *Mavs^−/−^* MDA5 peaks on a bp-wise basis), and one listing peaks present in both *Adar^−/−^; Mavs^−/−^* and *Mavs^−/−^*; again, only those peaks that were at least 10 bp long were kept.

For the control, control replicates were joined by retaining only those peaks that were within 200 bp of at least one peak in another replicate, and any peak shorter than 10 bp was removed.

RepeatMasker analysis was used to characterize repetitive elements found in the mapped reads. Finally, strand-specific ChipSeeker [40] annotation was performed using the Gencode VM23 (mm10) known gene data [41] to obtain a priority list of genomic regions [5′UTR, 3′UTR, Exon, Intron, Intergenic].

### RNA editing analysis in dsRNAs

Putative dsRNAs were detected using the latest RNA-seq curated annotations (downloaded from UCSC), according to the approach by Barak *et al.* [42]. Specifically, non-overlapping gene loci were selected, and for each locus, the longest mature transcript was extracted and aligned with itself by BLAST. Next, plus/minus hits were collected as putative dsRNAs. For each RNA-seq sample, A-to-I editing events falling in dsRNAs longer than 70 bases and with identity higher than 70% were identified by REDItools [43, 44], filtering out known SNPs from dbSNP (version 142 from UCSC). RepeatMasker (downloaded from UCSC) was used to annotate individual dsRNAs, while the editing activity per dsRNA, or the dsRNA editing activity per transcript, was calculated likewise the *Alu Editing Index* [24, 45].

### Cloning and *in vitro* transcription of protected RNAs

Protected peaks from ADAR1-deficient backgrounds contain many repeated sequences such as SINEs, LINEs, and LTRs. We selected a few top-enriched substrates from each category that also form strong dsRNA structures (predicted using the Vienna RNA fold web server). Using mouse gDNA as a template, these substrates were amplified with Q5 DNA Polymerase (Thermo Scientific) and cloned into the pGEM-T Easy Vector (Promega) according to the manufacturer’s instructions. Clones were confirmed by restriction digestion and sequencing with a T7 promoter primer. For *in vitro* transcription (IVT), the clones were linearized and gel-purified. The FAFAG-T7 RNA polymerase was used to synthesize the RNA using a standard T7 polymerase buffer (Thermo Fisher). The RNA was then dephosphorylated (5′ phosphate) with alkaline phosphatase (NEB) for 1 h at 37°C. RNA extraction was performed by using 2× PCI, 1× chloroform, and 96% ethanol. RNA quality was assessed by loading 1 µg of RNA onto a 2% agarose gel (5% formaldehyde) alongside the RNA RiboRuler.

### Cloning and purification of mutant, recombinant T7 RNA polymerase

To prevent the production of immunogenic dsRNA, if any, during *in vitro* transcription with T7 RNA polymerase, we introduced the G47A and 884G mutations into the *T7 RNA polymerase* gene, as described by Dousis *et al.* [46]. A clone encoding T7 polymerase with the critical mutations preventing loop-back transcription expressed in pBAD-6xHis was ordered from VectorBuilder (Neu-Isenburg, Germany). The mutant T7 RNA polymerase gene was subsequently expressed in *E. coli* BL21 (DE3) Rosetta cells. Protein expression was induced with 0.1 mM IPTG at 20°C overnight.

The cells were harvested and resuspended in lysis buffer containing 20 mM Tris–HCl (pH 8.0), 500 mM NaCl, 2 mM DTT, 10% glycerol, 1 mM PMSF, 0.1% Triton X-100, and 10 mM imidazole. Cell lysis was performed by adding 1 mg/ml lysozyme for 30 min at 25°C, followed by treatment with 10 µg/ml DNase I for 30 min at 25°C, and sonication. The lysate was then centrifuged at 30 000 rpm for 20 min at 4°C and filtered through a 0.45 µm sterile filter prior to loading onto a Ni-NTA column for purification. Purification of the recombinant protein was achieved using Ni-NTA affinity chromatography with an imidazole gradient. The protein was then dialyzed in buffer containing 40 mM Tris–HCl (pH 8.0), 25 mM NaCl, 5 mM DTT, 8 mM MgCl₂, and 2 mM spermidine hydrochloride. The quality of the purified protein was assessed by SDS–PAGE, and reaction conditions were optimized using various concentrations of protein and DNA templates.

### Inosine incorporation by IVT

To incorporate inosine into the RNA, we added ITP to the IVT reaction mixture and reduced the GTP concentration. To achieve ∼6% inosine-containing RNA, 1 mM ITP and 9 mM GTP were used. For ∼10% inosine incorporation, 2 mM ITP and 8 mM GTP were added. For ∼35% inosine incorporation, 5 mM ITP and 5 mM GTP were used. The remaining nucleotide concentrations were kept as recommended by the HiScribe™ T7 High Yield RNA Synthesis Kit, with 10 mM CTP, 10 mM UTP, and 10 mM ATP. The percentage of inosine incorporation in the RNA was subsequently quantified by mass spectrometry (Supplementary Fig. S7).

### *In vitro* A-to-I editing of dsRNA with ADAR proteins

To achieve A-to-I editing on dsRNAs, we used recombinantly purified ADAR2, ADAR1p110, and ADAR1p150 proteins for *in vitro* editing reactions. First, we optimized conditions for 100 nM of RNA. Briefly, 100 nM RNA-containing tubes were preheated in an editing buffer containing 30 mM HEPES (pH 7.5), 30 mM KCl, 15 mM NaCl, 15% glycerol, 2 mM MgCl₂, 1 mM EDTA at 70°C for 3 min, and then kept at room temperature for 10 min to refold. 1 mM DTT, 0.2 mM ATP, 0.5 μl of RNase inhibitor mix were added later to the reactions. The first tube contained double the amount of RNA and buffer mix. Next, we added 8000 nM ADAR protein to the first tube. Serial dilutions were performed to obtain protein concentrations ranging from 4000 nM to 100 nM (1:40–1:1) to determine the sufficient concentration for fully edited RNA. We found that 1500 nM ADAR2 protein (1:15 dilution) was sufficient to fully edit 100 nM dsRNA in 40 min at 37°C. In contrast, 4000 nM ADAR1p110 and ADAR1p150 proteins (1:40 dilution) were needed to fully edit the dsRNA in 2 h at 37°C. Subsequently, the volume of the RNA-editing reaction was increased to 250 µl with TE, and SDS was added to a final concentration of 0.1%. Subsequently, phenol (200 µl) was added, and the mixture was incubated on a thermo shaker for 3 min at 50°C. Following centrifugation, the aqueous phase was recovered and further purified by two consecutive phenol–chloroform, and chloroform extractions and subsequent precipitation. After precipitation, the RNA was resuspended in water, and an aliquot was run on a formaldehyde-agarose gel to check for RNA integrity (see Supplementary Fig. S8).

### Folding prediction of inosine-containing RNAs

Folding predictions of inosine-containing RNAs were generated using the Vienna RNA package [47].

### Overexpression of specific ADARs in editing free cells

To analyze the editing sites introduced into endogenous transcripts of interest by different ADARs, we overexpressed ADARs in editing-free, immortalized, MEFs [23] by two different means: ADAR2 was transiently transfected: 0.7 × 10^6^ Adar-free MEFs were electroporated with 5 μg of plasmid DNA using the NEON electroporation system (Thermo Fisher Scientific, Waltham, MA) with the following parameters: 1350 V, 30 mS, 1 pulse in a 100 μl NEON tip. The plasmid used was pCDNA3-ratAdar2-FLAG [48]. For Adar1p110 and Adar1p150, lentiviral plasmids (generously provided by the Carl Walkley lab) were packaged into virus and transduced into editing-free MEFs: pLVX-SM1 Adar1p110 3xFlag BclI Cw170418 and pLVX-SM1 Adar1p150 kozak 3xFlag NruI CW170418 were packaged using 10 µg of the pLVX-puro plasmids + 6 µg PS pax trono (Addgene #12260) and 6 µg pMD2.G VSVG packaging plasmids into 200 µl of 150 mM NaCl [49]. After adding 70 µl linear PEI, the mix was incubated for 10 min at RT, and then 1800 µl medium containing 10% FBS was added. This mix was added to 70% confluent Hek293 LX cells and incubated for 1.2 h in the incubator, followed by a medium change. Forty-eight hours later, viral supernatant was harvested, and editing-free MEFs were infected with viruses containing polybrene 5 µg/ml. Twenty-four hours later, selection with 5 µg/ml puromycin was started for 10–14 days, and expression of Adar1 p110 and p150 was verified by western blots probed with FLAG antibody (F7425, Sigma Aldrich, Merck, USA). Total RNA was extracted for editing analysis from cells 24 h post-transfection (ADAR2) or from a fully confluent plate of cells stably expressing ADAR1.

### cDNA synthesis and A-to-I editing detection

cDNA synthesis was performed using the LunaScript RT Master Mix Kit (NEB, primer-free). RNA was mixed with 6 µM of random hexamers, 5 µM of oligo dT, and nuclease-free water. The mixture was heated at 70°C for 4 min and then kept at room temperature for 10 min to anneal the primers. Next, 4 µl of LunaScript RT Master Mix (5X) was added, and reverse transcription was performed at 60°C for 40 min, followed by heat inactivation at 95°C for 1 min. To amplify the target, sequence-specific primers were designed to amplify short fragments of 250–300 bp. The amplified PCR product was run on a 1% agarose gel, and the bands were gel-purified using a gel elution kit. Sequencing was performed with forward and reverse primers of the target. The editing percentage was calculated based on the peak heights of A to G or C to T conversions.

### MDA5 ATPase assay

In a total reaction volume of 50 μl, 25 μl of an RNA containing 50 ng of RNA dissolved in buffer (20 mM HEPES, pH 7.5; 2 mM MgCl₂; and 150 mM NaCl) was heated at 70°C for 3 min, followed by refolding at room temperature for 15 min. Next, 25 μl of an MDA5 protein mix (containing 1 μg of MDA5, 2 mM DTT, 2 mM ATP, 20 mM HEPES, pH 7.5; 2 mM MgCl₂; and 150 mM NaCl) was added to the RNA mix. The mixture was gently mixed and incubated at 37°C for 15 min. The final RNA concentrations varied between 1.5 and 2 nM. To quench the reaction, 20 μl of 0.5 M EDTA was added, bringing the total volume to 70 μl. The level of released phosphate was measured by mixing 7 μl of the reaction mix with 200 μl Malachite Green (BioMol Green) and incubating at room temperature for 20 min. Optical density was measured at 650 nm (OD₆₅₀).

### MDA5 filament formation assay

In a total reaction volume of 50 μl, 25 μl of an RNA mix containing 50 ng of RNA dissolved in buffer (20 mM HEPES, pH 7.5; 2 mM MgCl₂; and 150 mM NaCl) was heated at 70°C for 3 min, followed by refolding at room temperature for 15 min. Next, 25 μl of an MDA5 protein mix (containing 1 μg of MDA5, 2 mM DTT, 2 mM AMP-PNP, 20 mM HEPES, pH 7.5; 2 mM MgCl₂; and 150 mM NaCl) was added to the RNA mix. The mixture was gently mixed and incubated at 22°C for 10 min. The final RNA concentrations varied between 1.5 and 2 nM. For electron microscopy analysis, Formvar-coated copper mesh grids were carbon-coated (2 nm) using a Leica ACE 200. A 5-μl aliquot of the sample was pipetted onto the grid and allowed to dry for ~60 s. The samples were then stained with one drop (∼5 μl) of 1% uranyl acetate (in water) for 30 s, followed by absorption with filter paper. This staining procedure was repeated using a 2% uranyl acetate solution. Finally, the samples were dried at room temperature and stored in the dark at 4°C. TEM analysis was performed using a Tecnai G2 20 transmission electron microscope (FEI Company, USA) operating at 80 kV, equipped with an FEI Eagle 4K CCD camera.

### RNA transfections

3*10^5^ MEFs were seeded per well of a 12-well cell culture dish and transfected with 300 ng of refolded RNAs using Lipofectamine 3000 (Invitrogen) according to manufacturer’s protocol. Cells were incubated for 24 h, followed by RNA extraction for RT-qPCR or staining for immunofluorescence imaging of stress granules. For co-transfection experiments, 250 ng of refolded dsRNAs (Rnf168 and Zkscan) or 250 ng of polyI:C plus 80 pmol of annealed short ds-RNA oligos (either containing one U-A plus three C–G base pairs or four I–U pairs in the middle of a short ds oligo pair as described [50]) were transfected and analyzed by RT-qPCR. For stress granule quantification, 3*10^4^ MEFs per well of a 12-well dish were seeded and stained for immunofluorescence imaging of stress granules.

### RT-qPCR

Total RNA was extracted using TRIzol reagent, DNAseI treated (New England Biolabs, Ipswich, MA), and cDNA was synthesized using LunaScript^®^ RT SuperMix Kit (NEB) according to the manufacture’s instruction. Real-time PCR was performed using a Luna^®^ Universal qPCR Master Mix (NEB) and the primers (see Supplementary Table S2) using a BioRad CFX machine. Mouse actin mRNA was used as the internal normalization control. ISG expression was calculated using the ΔΔCq method comparing different RNA transfections to the mock control (with water instead of RNA).

### Stress granule formation

Formation of stress granules 24 h post-RNA transfection was assessed with immunofluorescence staining using an anti-G3BP1 antibody (ab56574, Abcam) in combination with Alexa 488 secondary antibody (Invitrogen, Thermo-Fisher Scientific, Waltham, MA) and mounted in Prolong Diamond antifade solution with DAPI (Life Technologies). Microscopic confocal sections were taken on a confocal laser scanning microscope FV3000 (Olympus, Tokyo, Japan). Images were processed with ImageJ and AffinityPhoto. Quantification of stress granules was done from at least three independent transfections and three 40× images per condition. Number of cells with granules/total cell number was counted.

### Statistical evaluation

*For stress granule formation*, quantification was performed from at least three independent transfections, analyzing at least nine images of 100 µm × 100 µm per condition. The proportion of cells with granules relative to the total cell count was calculated for each condition. Mean number of stress granules, standard deviation, and T test were performed using GraphPad Prism. ATPase assays and qPCR assays for testing the expression of interferon-stimulated genes were performed in triplicate. Mean, standard deviation, and T-test were performed in GraphPad Prism.

### ADAR cloning and expression

The coding sequence of human ADAR1p110 (p110, Uniprot P55265-5) was cloned with N-terminal TwinStrep and C-terminal His6 tags into a pCAGGS vector while the ADAR1p150 (Uniprot P55265-1) with N-terminal truncation of 2-133 amino acids (Δ133 p150) was cloned with N-terminal His6 and C-terminal TwinStrep tags in the same vector. A 3C protease site was placed between the gene and affinity tags. Recombinant ADAR1 was transiently expressed in the Expi293F cells following the Expi293 expression system user guide (Thermo Scientific, Publication Number MAN0019402). Cell transfection was carried out with PEI (25-kDa linear polyethyleneimine, Thermo Scientific) instead of Expifectamine 293 reagent. Transfected cells were cultured in suspension for 42–50 h, harvested by centrifugation, and stored at −80°C for later use.

The ADAR2 coding sequence (Uniprot P78563-2) was cloned into a modified, pFastBac-derived 438-B vector [51] containing an N-terminal His_6_ tag followed by a 3C protease site. Bacmids (from electroporation into DH10EMBacY cells, Geneva Biotech) and V1 baculovirus (from transfection into Sf9 cells, Thermo Fisher Scientific) were generated according to published protocols [52]. For protein expression, High Five insect cells (Thermo Fisher Scientific) were grown in ESF 921 medium (Oxford Expression Technologies), infected with ADAR2-containing V1 baculovirus, and grown for 48–72 h at 27°C. Cells were harvested by centrifugation and stored at −80°C.

### ADAR1 purification

The purification procedure was carried out at 4°C, and the same protocol was used to purify both ADAR1 isoforms. The thawed cell pellet was lysed by sonication in buffer 1 [50 mM HEPES-NaOH pH 8.0, 500 mM NaCl, 20% (v/v) glycerol, 1 mM DTT] supplemented with 1× protease inhibitor (1 mM PMSF, 2 mM benzamidine, 1 μM leupeptin, and 2 μM pepstatin). The cell lysate was clarified by centrifugation followed by filtration through a 0.45 μm syringe filter and loaded onto a 5-ml HisTrap excel column (Cytiva) equilibrated with buffer 1. The unbound proteins were washed with buffer 2 [50 mM HEPES-NaOH pH 8.0, 500 mM NaCl, 20% (v/v) glycerol, 5 mM EDTA, 1 mM DTT] containing 50 mM imidazole. To remove contaminating chaperones, the column was washed with buffer 1 containing 4 mM ATP-MgSO_4_. The protein was eluted with buffer 2 containing imidazole with a linear gradient from 50 mM to 500 mM imidazole. Eluted protein was loaded onto a 1-ml StrepTrap HP column (Cytiva) equilibrated with buffer 2 followed by a column wash with the same buffer. Protein was eluted with buffer 3 [50 mM HEPES-NaOH pH 8.0, 500 mM NaCl, 20% (v/v) glycerol, 1 mM EDTA, 1 mM DTT, 2.5 mM d-Desthiobiotin]. The affinity purification tags were removed by protein incubation with 3C PreScission Protease. Cleaved protein was loaded onto a HiLoad 16/600 Superdex 200 pg column (Cytiva) equilibrated with ADAR1 SEC buffer [50 mM HEPES-NaOH pH 8.0, 350 mM NaCl, 15% (v/v) glycerol, 0.5 mM EDTA, 1 mM DTT]. The elution peak was concentrated using an Amicon centrifugal filter with a 50 kDa cut-off membrane (Millipore) to a concentration of 4–5 mg/ml. Protein was aliquoted, flash-frozen in liquid nitrogen, and stored at −80°C until use. The yield was ~500 µg per L of culture.

### ADAR2 purification

All steps were carried out at 4°C. Insect cell pellets from the expression of ADAR2 were lysed by sonication in buffer A [50 mM HEPES pH 7.8, 500 mM NaCl, 10% (v/v) glycerol, 1 mM DTT] supplemented with 10 mM imidazole and 1× protease inhibitor (1 mM PMSF, 2 mM benzamidine, 1 μM leupeptin, and 2 μM pepstatin). Lysate was clarified by centrifugation and ultracentrifugation followed by filtration through a 0.45-µm filter and applied to a 5-ml HisTrap HP column (Cytivia). The column was washed with buffer A supplemented with 50 mM imidazole and with high salt buffer [50 mM HEPES pH 7.8, 1 M NaCl, 10% (v/v) glycerol, 1 mM DTT]. Protein was eluted with buffer B [50 mM HEPES pH 7.8, 150 mM NaCl, 10% (v/v) glycerol, 1 mM DTT, 250 mM imidazole]. The eluate was applied to a 5-ml HiTrap Heparin column (Cytivia) and eluted with a linear gradient from 0.15 M to 1 M NaCl in buffer C [50 mM HEPES pH 7.8, 10% (v/v) glycerol, 1 mM DTT]. The ADAR2-containing fractions were pooled and cleaved overnight with 3C protease. The cleaved protein was passed over the 5-ml HisTrap HP column in buffer A and eluted from the column with buffer A supplemented with 50 mM imidazole. The pooled protein was split into aliquots and each was subjected to chromatography on a Superdex 200 Increase 10/300 GL column (Cytivia) equilibrated in ADAR2 SEC buffer [20 mM HEPES pH 7.8, 300 mM NaCl, 10% (v/v) glycerol, 1 mM DTT]. Peak fractions were combined and concentrated using a 30-kDa cutoff Amicon spin concentrator (EMD Millipore) to a concentration of ~10 mg/ml, flash-frozen in liquid nitrogen, and stored at −80°C until use. Yield was ~7 mg per L of culture.

## Results

### MDA5 binds repeat elements in endogenous transcripts

To identify RNAs that are bound by MDA5 in the absence of ADAR1-mediated editing we modified a protocol previously applied to identify human RNAs that can be bound by MDA5. Briefly, total RNA from liver of 8-day-old ADAR1-deficient mice, rescued by lack of MAVS (*Adar*^−/−^; *Mavs*^−/−^), and *Mavs*^−/−^ mice as a control were incubated with recombinantly produced mouse MDA5^ΔCARD^, a deletion version of MDA5 from which the CARD domains had been removed to facilitate expression [53]. Unbound RNAs were digested using an RNaseA/T1 mix. Protected RNAs from MAVS-deficient (*Mavs*^−/−^) and editing-deficient (*Adar*^−/−^; *Mavs*^−/−^) mice were used to generate NGS libraries. RNase-resistant sites were identified in samples treated with RNases in the absence of MDA5 ^ΔCARD^ [54]. Libraries generated from total input RNAs were used to determine RNA abundancies (Supplementary Fig. S1). All RNA extractions, library preps, and sequencing were performed in biological triplicates. Libraries were sequenced 75 bp single-end, ranging between ∼30 m reads (input) to ∼10 m reads for non-protected RNase-treated samples (see Supplementary Table S1). Principal component analysis shows a clear clustering of libraries generated from *Adar*^−/−^; *Mavs^−/−^* versus *Mavs*^−/−^ livers for both input and MDA5 ^ΔCARD^-protected samples (Supplementary Fig. S2).

**Figure 1.**
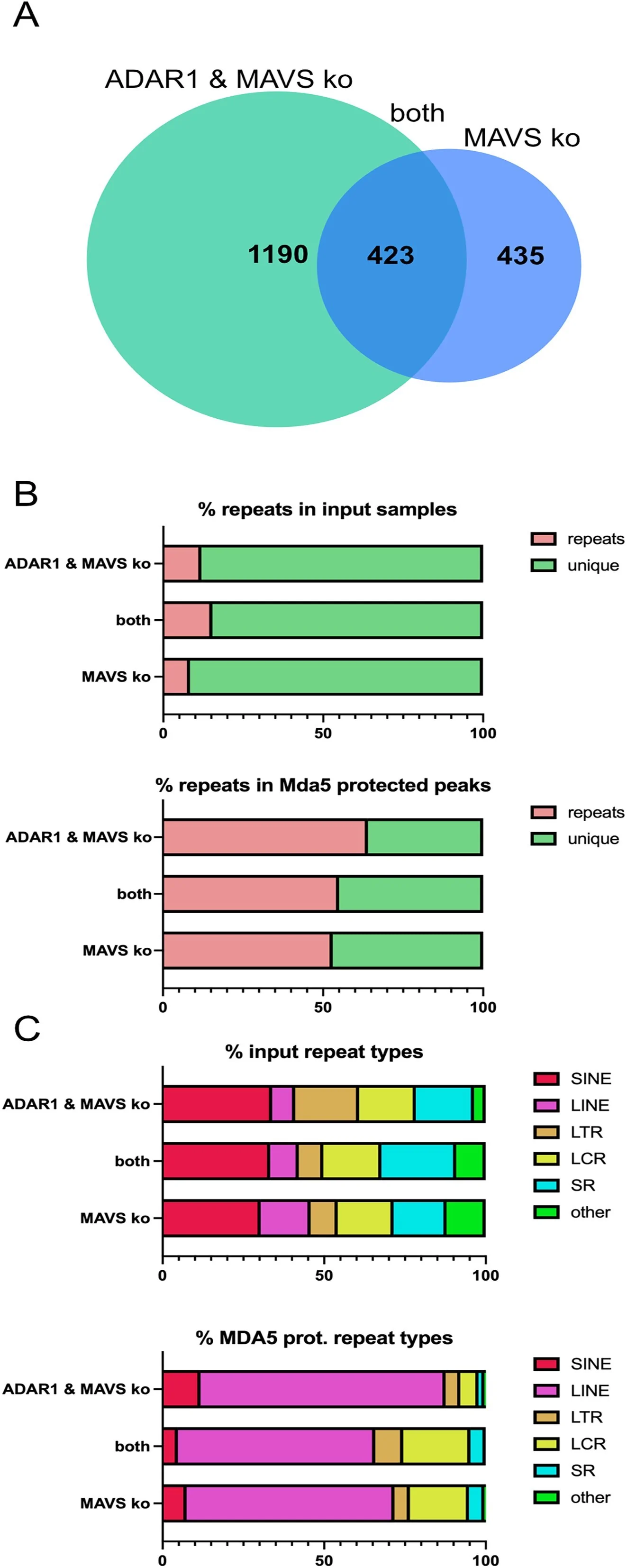
MDA5 binds repeat elements in endogenous transcripts. (**A**) Total liver RNA from ADAR1 knockout (Adar^−/−^; Mavs^−/−^) or MAVS-deficient (Mavs^−/−^) mice was incubated with MDA5 and digested with RNaseA. Protected RNAs were used for NGS library generation and sequencing. Venn diagram showing the number of RNA peaks identified in ADAR1 knockout (Adar^−/−^; Mavs^−/−^) and MAVS-deficient (Mavs^−/−^) samples following RNase protection, sequencing, and peak calling using Genrich. A total of 1190 peaks were uniquely protected by MDA5 in ADAR1- & MAVS-deficient RNAs, while 435 peaks were uniquely protected in MAVS-deficient RNAs. Additionally, 423 peaks were common to both samples. (**B**) Stacked bar plot showing the results of RepeatMasker analysis for input samples and MDA5-protected peaks, illustrating the percentage of overlap with repetitive elements. Overall, the percentage of repetitive elements is significantly higher in MDA5-protected samples compared to their respective input samples. In the Adar^−/−^; Mavs^−/−^ sample, ~64% (760 peaks) of MDA5-protected peaks overlap with repeats, while only 13% (766 peaks) of input sequences contain repeats. (**C**) Stacked bar plot depicting the distribution of repeat element classes in input sequences and MDA5-protected peaks. In input samples, SINEs make up the largest portion, while in MDA5-protected repeats, a large fraction overlaps with LINEs (61%–76%), followed by SINEs (7%–12%) and LTRs (∼5%), depending on genotype. Input sequences show a higher SINE content (30%–34%) and a lower LINE content (15%–7%). The relative fraction of SINEs is higher (12%) in Adar^−/−^; Mavs^−/−^ samples relative to 7% in Mavs^−/−^ samples.

Peaks were called in all samples using *Genrich* with the input samples as control and an adjusted *P*-value of .01. Protected peaks were only considered if found in ≥ 2 samples. Peaks were considered individual peaks if separated ≥ 200 nt apart from each other. RNA fragments that were resistant to RNase digestion in the absence of MDA5 were subtracted from the MDA5-protected samples. Also, peaks shorter than 10 bp were excluded from further analysis. Following this pipeline, 1190 peaks were uniquely found protected by MDA5 in the ADAR-deficient RNAs, while 435 peaks were found uniquely protected in MAVS-deficient RNAs. 423 peaks were found common in both samples (Fig. 1A).

As expected, a large fraction of MDA5-protected reads (∼63%, 749 peaks) specific to *Adar*^−/−^; *Mavs^−/−^* overlap with repeats. In contrast, the input sequences had a 12% (717 peaks) repeat content in the *Adar*^−/−^; *Mavs^−/−^* sample (Fig. 1B). When comparing the occurrences of these repeats, it becomes obvious that MDA5-protected repeats locate mostly to 3′ UTRs, introns, and intergenic regions (Supplementary Fig. S3). Amongst the MDA5-protected repeat fraction, a large fraction overlaps with LINEs (67%–76%), SINEs (8%–12%), and LTRs (∼5%), depending on the genotype. Interestingly, the input sequences had a much higher SINE content of 30%–34%, while the fraction of LINEs was comparatively low (7%–15%), depending on the genotype (Fig. 1C and Supplementary Data File 2). When comparing the MDA5-protected peaks specific to MAVS-deficient (*Mavs^−/−^*) with those specific to ADAR1 deletion mice (*Adar*^−/−^; *Mavs^−/^*^−^), the strongest relative increase was observed for the SINE fraction (8% in *Mavs^−/−^* versus 12% in *Adar*^−/−^; *Mavs^−/^*^−^). This is consistent with previous reports that demonstrated that SINEs can activate Mda5 in certain epigenetic cancer therapies [55]. We also tested whether the length of the MDA5 protected regions would correlate with the enrichment *P*-value of the protected region. To do so, we considered all repeats, as well as SINEs and LINEs separately. Interestingly, the length of the protected region did not correlate well with the significance of enrichment. There was also no major difference to be seen between protected regions derived from SINEs and those derived from LINEs. However, there was an average increase in the median length of MDA5^ΔCARD^ protected peaks between RNAs from MAVS-deficient and ADAR1 & MAVS-deficient mice. This clearly shows that in the absence of ADAR1, longer regions can be bound by MDA5 ^ΔCARD^ (Supplementary Fig. 4A and B). This finding is true for both repeat-containing peaks and also for peaks found outside of repeats. The median length of protected peaks is around 158 nt for editing-deficient (*Adar^−/−^; Mavs^−/−^*) samples, while it is only 90 nt for editing-competent *Mavs*^−/−^ samples. Since the peaks found exclusively in the ADAR-deficient sample and those exclusive to the *Mavs*^−/−^ samples did not overlap, we directly compared peak lengths of peaks found in both *Adar^−/−^; Mavs^−/−^* and *Mavs*^−/−^ samples. Also here, the median peak length was at 190 nt for ADAR-deficient samples, while it was at 117 nt in the control (*Mavs*^−/−^) sample (Supplementary Fig. S4C). The different lengths of protected RNAs can certainly explain why filaments formed by MDA5 are on average longer in ADAR-deficient samples. However, given that the longest protected peaks are still in the range of 800–1000 nt in the wild-type (*Mavs*^−/−^) samples, it also suggests that, in principle, longer filaments might be formed by MDA5, even in editing competent tissues.

Recently, natural antisense transcripts had been reported to trigger MDA5 activation in the absence of editing [56]. We therefore specifically intersected the MDA5-protected fraction from *Adar*^−/−^; *Mavs^−/^*^−^ mice with the cisNATs identified in the GencodeVm23 annotation. However, this resulted in a single overlap of a 63 bp-long region on chromosome 13 and was therefore not further considered for additional analyses. Still, to identify RNAs with the potential to elicit an IFN response in the absence of ADAR1, we compiled all 1190 MDA5-protected peaks that are unique to *Adar*^−/−^; *Mavs^−/^*^−^ mice and calculated a minimal and maximal adjusted *P*-value considering their expression and fold enrichment in the MDA5 ^ΔCARD^-protected *Adar*^−/−^; *Mavs^−/^*^−^ sample. Moreover, genomic annotation and potential repeat origin were included (Supplementary Data File 2). Intronic repeat-derived sequences had been shown to trigger an antiviral interferon response. However, this was primarily seen in combination with specific chromatin mutations or upon splicing inhibition [57, 58]. In our case, we had used total RNA from ADAR-deficient mice, which show only minor splicing defects [59]. We therefore reasoned that the MDA5 ^ΔCARD^-protected intronic regions are a result of using total cellular RNAs, including unspliced pre-mRNAs, which would be unlikely to activate the cytoplasmic dsRNA sensor MDA5 *in vivo*. We also determined the average RNA editing ratio (RNA-editing index) of protected peaks for protected SINEs, LINEs, LTR elements, and the sum of all repeats [24]. This was done on mRNAs derived from wild-type cells and cells selectively lacking ADAR1p150 [12] (Supplementary Fig. S5). Both SINEs and LTR elements showed a clear, ADAR1p150-specific editing rate, while LINE elements showed hardly any RNA editing. Taken together, we therefore reasoned that SINEs and LTR elements would be the major source of dsRNAs that can trigger MDA5 activation. Based on this assumption, we picked two SINE-derived peaks (*Rnf168* chr16:32300187-32300400, *Knl1* chr2:119104854-119104978) and cloned them with their adjacent, inverted SINE element. We also cloned the fragment corresponding to one peak derived from an LTR element (*Zkscan3* chr13:21392247-21392435) and a LINE-derived fragment (chr15:82697668-82699436) into pGEM-T easy, which allows the generation of RNA transcripts by T7 RNA polymerase. For comparison, we also included the 3′ UTR of the human zinc finger-encoding *ZNF-708* transcript, which contains two inverted Alu elements and can form an extended secondary structure. *In silico* folding and generation of a centroid structure using the ViennaRNA package indicates that the 3′ UTRs of *Rnf168, Knl1, Zkscan3*, and *ZNF-708* can form extended double-stranded structures of ∼160, 220, 250, and 300 bp, respectively. In contrast, the LINE-derived fragment seemingly cannot fold an extended double-stranded structure (Supplementary Fig. S6).

To test whether these RNAs would indeed be able to support MDA5 ^ΔCARD^ polymerization, which would lead to subsequent activation of MAVS and initiate an interferon response, we made T7 *in vitro* transcripts of the cloned regions of *Rnf168, Knl1, Zkscan3*, and the cloned LINE element. As a positive control, we also included the 3′ UTR of the human *ZNF-708* gene, which harbors two Alu elements. As a negative control, we used only half of the Rnf168 UTR, which only harbors a single SINE element and therefore should not form an extended double-stranded structure.

**Figure 2.**
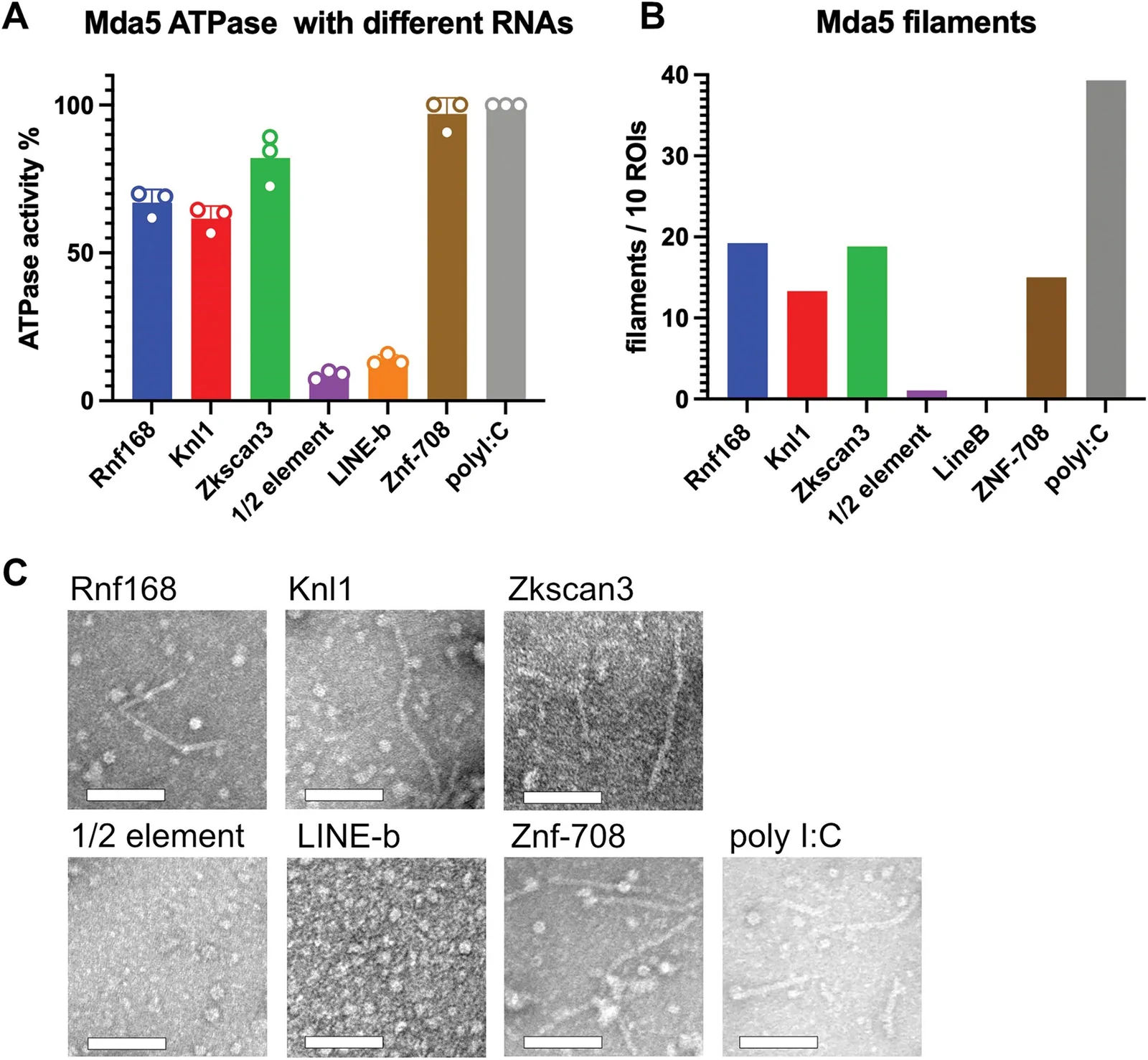
Structured, MDA5-protected RNAs support MDA5 polymerization. (**A**) *In vitro* transcribed RNAs harboring two inverted SINEs from the 3′ UTRs of *Rnf168* and *Knl1*, an LTR element (*Zkscan3*), a single SINE (1/2 element), or a LINE-b element were tested for their ability to support ATP hydrolysis of MDA5. The 3′ UTR of the human *ZNF-708* transcript, harboring two inverted Alu elements, and poly I:C were used as controls. Indeed, the inverted SINEs and the LTR element do efficiently support ATP hydrolysis, while a single SINE (1/2 element) and a LINE-b element fail to support ATP hydrolysis. (**B**) In the presence of non-hydrolyzable AMP-PNP, MDA5 forms filaments along the structured 3′ UTRs of Rnf168, Knl1, and the LTR element in *Zkscan3*, similar to *ZNF-708* and poly I:C. Again, the single SINE and the LINE-b element fail to support filament formation. (**C**) Electron micrographs showing the formation of filaments on the inverted SINEs found in the 3′ UTRs of *Rnf168, Knl1*, or the LTR element *Zkscan3*. Human *ZNF-708* harboring two inverted Alu elements and poly I:C RNA serve as positive controls. Bar = 100 nm.

T7 RNA polymerase can do foldback transcription, leading to inadvertent production of dsRNAs [60]. Therefore, to avoid untemplated dsRNA production, we took advantage of a recently generated mutant version of T7 polymerase, which strongly reduces non-templated loopback transcription [46]. Briefly, a His-tagged T7 clone was synthesized to carry the critical G474A amino acid exchange and terminal 884G addition. Following the addition of the C-terminal G, we call this T7 polymerase T7_FAFAG_. RNA produced with this polymerase was used to test for MDA5 polymerization. We generated a mouse MDA5^ΔCARD^ clone covering aa 231 to 1025 that deletes the N-terminal CARD domains. The construct was C-terminally His-tagged, allowing bacterial expression and purification. To determine the ability to support MDA5 to bind and proofread these RNAs, we performed an ATPase assay, which colorimetrically measures ATP hydrolysis that occurs during MDA5 binding and proofreading of dsRNAs [3, 61]. Poly I:C was used as a standard and set to 100%. These data clearly showed that the inverted SINEs found in *Rnf168* and *Knl1*, but also the secondary structure formed by the LTR located in Zkscan3, can support MDA5 ^ΔCARD^ binding and proofreading to a similar extent as poly I:C or the inverted Alu elements found in human *ZNF-708* (Fig. 2A). In contrast, a single SINE found in Rnf-168 but also the LINE-b element did not lead to ATP-hydrolysis, indicating that these unstructured RNAs do not support MDA5 ^ΔCARD^ dsRNA-binding and subsequent dsRNA-proofreading. We also tested the ability of Mda-5 filament formation by electron microscopy of Mda-5 ^ΔCARD^ filaments formed along different RNAs in the presence of non-hydrolysable AMP-PNP. Again, the dsRNAs formed by the 3′ UTRs of *Rnf168* or *Knl1*, but also the LTRs of *Zkscan3* did support polymer formation, as did the human inverted Alus found in *ZNF-708* or poly I:C. A single SINE found in *Rnf168* (1/2 element) or a LINE-b element did not support filament formation (Fig. 2C). Quantitation of filaments across 10 regions of interst (ROIs) confirmed these findings (Fig. 2B). The predicted double-stranded structures of *Rnf160, Knl1, Zkscan3*, and *ZNF-708* should form dsRNA structures in the range of 50 nm (*Rnf168*) to 100 nm *(ZNF-708*), respectively. Indeed, *Rnf168* gave rise to the shortest fragments of ∼70 nm, while *ZNF-708* showed the longest filaments of ∼120 nm. However, we also observed occasional filaments of 200 or 300 nm (see also Supplementary Figs S9 and S10). Such long structures might be the result of intermolecular head-to-tail annealing of two RNA molecules or partial base pairing and stacking of regions not accurately predicted by the ViennaRNAfold package.

### Inosines introduced by RNA editing but not by *in vitro* transcription repress MDA5 ΔCARD polymerization

*In vivo*, ADAR1 but not ADAR2 editing activity is critically required to suppress MDA5-mediated activation of the immune system [10]. Additionally, RNA-binding activity of ADAR1p150 but also protein–protein interactions suppress activation of the Z-DNA-binding protein ZBP and the dsRNA-dependent kinase PKR [15, 27, 28, 62–64]. ADARs can destabilize RNAs when an A:U base pair is converted into an I:U wobble base pair but can also stabilize RNAs when an A:C mismatch is changed into an I:C base pair. It has also been shown that short RNAs containing consecutive I:U basepairs can suppress IFN-signaling and distort the helicity of dsRNAs [50, 65, 66]. We therefore wanted to test whether the presence of inosines *per se* or a specific set of inosines introduced by ADARs might suppress MDA5 activation *in vitro* and *in vivo*. To test this, we transcribed single-stranded and dsRNAs using T7_FAFAG_ RNA polymerase. RNA-editing by ADARs deaminates adenosines into inosines. The resulting A to I exchange mimics an A to G exchange in many respects [67]. However, due to the propensity of inosines to basepair with cytosines, inosines can only replace guanosines during *in vitro* transcription. We therefore replaced GTP with ITP in different ratios during *in vitro* transcription. ITP levels above 50% strongly impaired RNA synthesis. We therefore determined the rate of inosine incorporation for RNAs synthesized in the presence of 50%, 20%, or 10% ITP to GTP. HPLC analysis of the hydrolyzed RNAs indicated that inosine incorporation was at ∼35%, ∼10%, and 6%, respectively, relative to G at the tested concentrations (Supplementary Fig. S7A–C). Thus, RNAs were generated that had inosines stochastically replacing guanosines, presumably equally distributed along the entire transcript. To compare this with RNAs that had inosines introduced by deamination of adenosines, we performed *in vitro* editing reactions on *in vitro* transcribed RNAs using recombinantly purified ADAR1p150, ADAR1p110, and ADAR2. By doing so, adenosines were converted to inosines in a non-stochastic manner. Successful deamination was determined by Sanger sequencing of RT-PCR products of the *in vitro* edited transcripts. In the sequence chromatograms, the ratio of Inosine (G) peaks over the sum of I (G) plus A peaks for all adenosines located in double-stranded structures was determined to calculate levels of deamination, seen as A-to-G conversion. Interestingly, we noticed that different sites are edited at different efficiencies. While some sites could be edited to almost 100%, others were only edited to low levels, even if those corresponded to the reported ADAR next-neighbor preference and despite extended incubation times with ADARs (see materials) [68]. This suggests that some sites might be inhibited by nearby, highly efficient editing events. Average *in vitro* editing levels for all adenosines within the mouse structured RNAs derived from SINEs (*Rnf168, Knl1*) and the LTR element in *Zkscan3* ranged from 6 to 14% (Supplementary Fig. S7D). The average editing level of all adenosines within the human-derived inverted Alu elements in *ZNF-708* even reached 26%. All three ADARs, ADAR1p150, p110, and ADAR2, gave rise to comparable editing levels.

**Figure 3.**
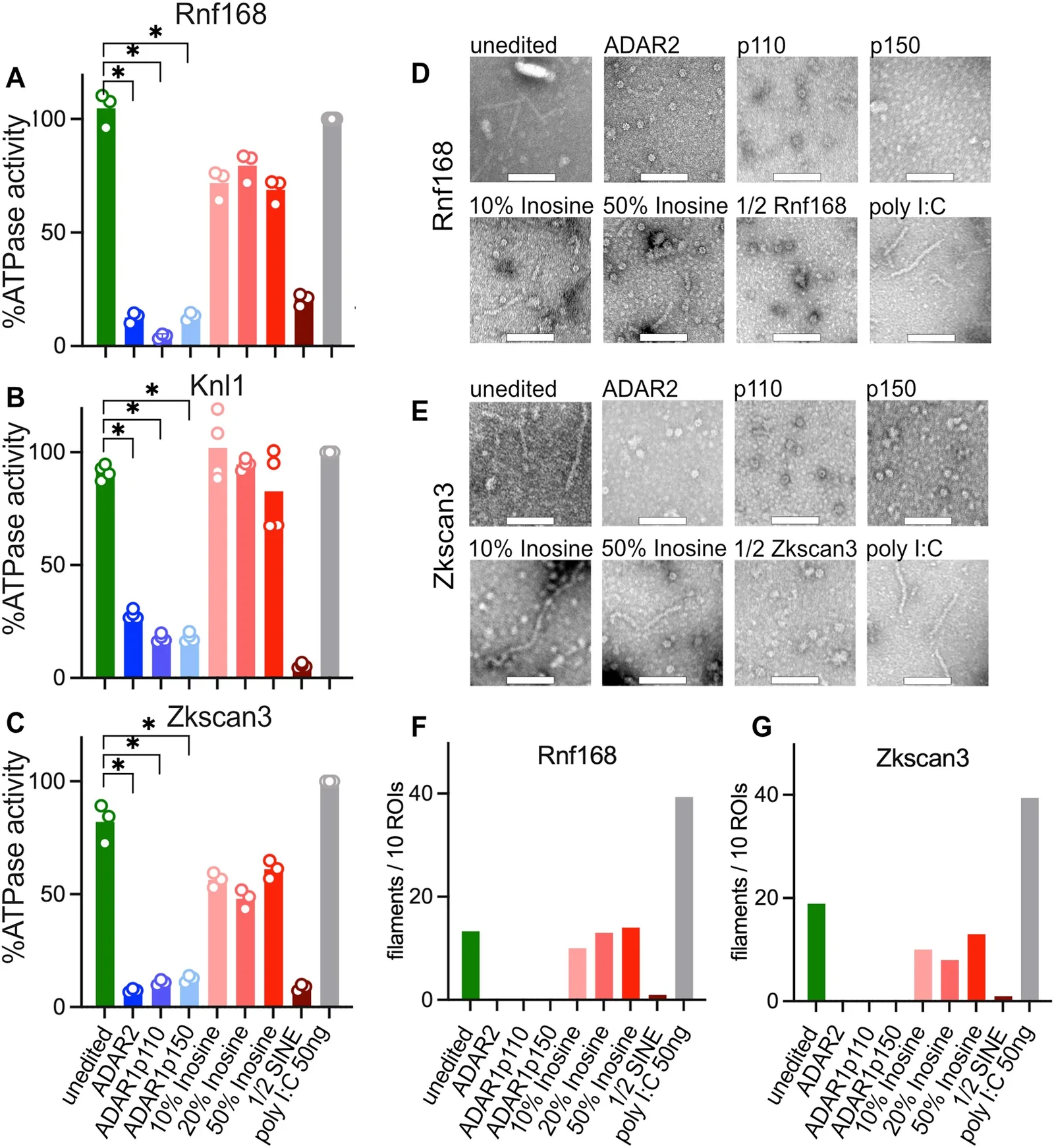
Inosines introduced by ADAR editing, but not by *in vitro* transcription, repress MDA5 polymerization. ATP hydrolysis assays as a readout of MDA5 polymerization on *in vitro*-transcribed RNAs of the inverted SINE-containing 3′ UTRs of Rnf168 (**A**) and Knl1 (**B**), or the LTR element Zkscan3 (**C**). RNAs were transcribed using T7_FAFAG_ RNA polymerase and edited *in vitro* with ADAR2, ADAR1p110, or ADAR1p150. Alternatively, GTP was substituted with ITP to 10%, 20%, or 50%. A single SINE (1/2 element) and poly I:C served as controls. For the ATP hydrolysis assay, refolded RNA was incubated with MDA5 and ATP. ATP hydrolysis was measured using a malachite green detection kit. RNAs with inosines incorporated during transcription failed to suppress ATP hydrolysis, while RNAs modified by *in vitro* editing with ADARs effectively prevented ATP hydrolysis. Notably, RNA containing the single SINE (1/2 element) did not induce ATP hydrolysis. * denotes *P* value < .001. (**D, E**) Electron micrographs showing the results of MDA5 filament formation assays in the presence of non-hydrolysable AMP-PNP, which stabilizes MDA5 filaments on dsRNA. Structured 3′ UTRs of RNF168 (D) and Zkscan3 (E). The RNAs were edited *in vitro* with ADAR2, ADAR1p110, or ADAR1p150. *In vitro* transcription was also performed in the presence of 10% or 50% ITP, substituting GTP. A single SINE of RNF (1/2 Rnf168) or a single-stranded region of Zkscan3 (1/2 Zkscan3), as well as poly I:C, were used as negative and positive controls, respectively. MDA5 filaments were spotted on carbon-coated grids, stained with uranyl acetate, and imaged by TEM. RNAs with inosines incorporated during transcription failed to suppress MDA5 polymerization, while RNAs modified by *in vitro* editing effectively prevented MDA5 oligomerization. Notably, RNA containing the single SINE (1/2 element) did not support MDA5 filament formation. Bar = 100 nm. (**F, G**) Bar graphs showing the results of MDA5 filament quantifications of 10 fields of 1 µm × 1 µm of the data shown in panels (D and E), respectively.

The RNAs generated this way were first tested for their integrity on RNA gels (Supplementary Fig. S8). RNAs were not affected in their integrity by the in vitro editing reaction. Also, as RNAs were running at a single band, we can exclude that ADAR would still be bound to the RNAs after phenol extraction and precipitation. Next, RNAs were tested for their ability to activate MDA5 polymerization *in vitro* by determining ATP hydrolysis and filament formation. Despite showing higher inosine rates, RNAs with inosine incorporated during transcription, and thus replacing guanosines, failed to suppress ATP hydrolysis or MDA5 polymerization. In contrast, however, RNAs modified by *in vitro* editing and therefore replacing adenosines by inosines, efficiently prevented ATP hydrolysis (Fig. 3A–C). Most surprisingly, while ADAR1p150 is critically required to suppress MDA5 activation *in vivo*, ATP hydrolysis by MDA5 ^ΔCARD^ was equally well suppressed by *in vitro* editing using ADAR1p150, ADAR1p110, or ADAR2. Likewise, MDA5 ^ΔCARD^ filament formation was also prevented by inosines introduced by editing using either ADAR isoform, but not by introducing inosines during *in vitro* transcription (Fig. 3D–G and Supplementary Figs S9 and S10).

### Only inosines introduced by *in vitro* editing can suppress IFN response in cells

**Figure 4.**
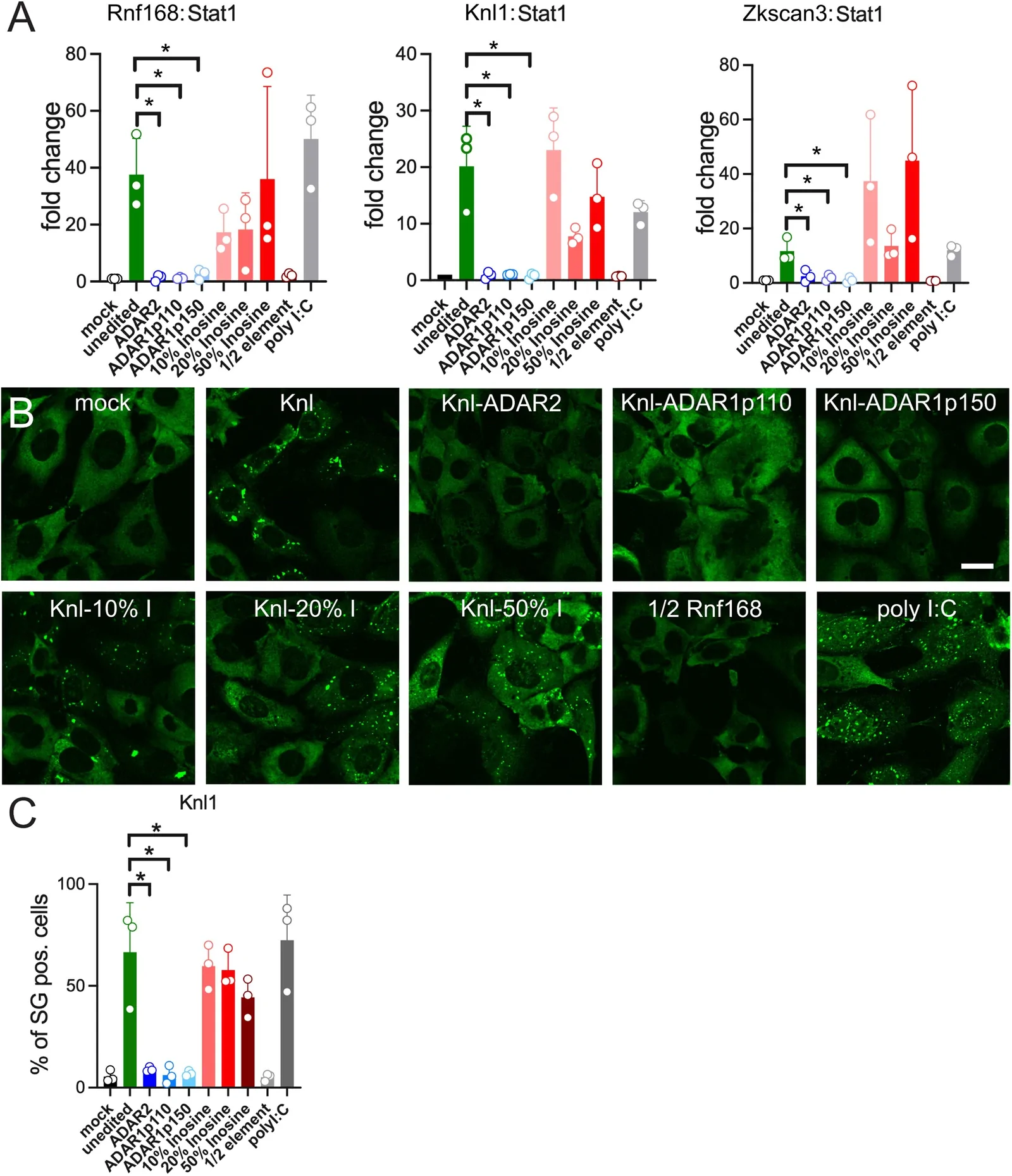
Inosines introduced by *in vitro* editing suppress IFN response and prevent stress granule formation. (**A**) Immortalized MEFs were transfected with 300 ng of *in vitro* transcribed, dephosphorylated, and refolded RNA derived from the 3′ UTRs of Rnf168, Knl1, or Zkscan3, or with the same RNAs edited *in vitro* using ADAR2, ADAR1p110, or ADAR1p150. Alternatively, GTP was substituted with ITP to 10%, 20%, or 50% during *in vitro* transcription. Subsequently, total RNA was extracted, and cDNA was synthesized from 200 ng of RNA. RT-qPCR was then performed to measure Stat1 expression, with GAPDH used as a reference. The fold change in expression relative to mock-transfected (transfection reagent only) controls is plotted on the Y-axis. Editing by ADAR2, ADAR1p110, or ADAR1p150 suppresses interferon induction, while inosines introduced during *in vitro* transcription fail to do so. Poly I:C RNA is used as a positive control. (**B**) Microscopy images showing stress granule formation 24 h post-RNA transfection with the RNAs described in panel (A). Immortalized MEFs were transfected and stress granule formation was assessed using immunofluorescence staining with an anti-G3BP1 antibody. Scale bar = 10 µm. (**C**) Bar graph quantifying stress granule formation. Quantification was performed from at least three independent transfections, analyzing at least nine images of 100 µm × 100 µm per condition. The proportion of cells with granules relative to the total cell count was calculated for each condition shown in panel (B).

MDA5 ^ΔCARD^ polymerization *in vitro* may not reflect the situation in cells. Also, the deletion of the CARD domains can affect the efficiency of polymerization [4, 69]. Therefore, native RNAs and RNAs that had inosines replacing guanosines introduced during *in vitro* transcription, or *in vitro* edited RNAs where adenosines are converted to inosines, were transfected into immortalized MEFs and tested for their ability to activate an IFN response. As the 5′ phosphate of *in vitro* transcribed RNAs can also activate RIG-I, leading to an IFN response, the *in vitro* transcribed RNAs were also dephosphorylated prior to transfection. IFN induction was monitored by qRT-PCR for the interferon-induced genes (ISGs) *Stat1* or *Irf7*. As expected, RNAs with a strong secondary structure, the 3′ UTR of *Rnf168, Knl1*, and the LTR element in *Zkscan*3, triggered expression of ISGs to a similar level as poly I:C, while a single SINE element (1/2 element) failed to do so (Fig. 4 and Supplementary Fig. S11).

When comparing RNAs with inosines introduced during *in vitro* transcription or by *in vitro* editing, only RNAs containing inosines introduced by *in vitro* editing were able to efficiently reduce IFN induction while RNAs with inosines introduced by *in vitro* transcription failed to do so (Fig. 4A and Supplementary Fig. S11). Most interestingly, RNAs edited by any of the three ADAR isoforms efficiently inhibited activation of MDA5^ΔCARD^  *in vitro* and prevented activation of IFN-stimulated genes in cells. To ensure that RNAs edited by ADARs *in vitro* do not contain trace amounts of recombinant ADAR that would interfere with the interferon activation upon transfection, we also treated poly I:C RNA with recombinant ADAR2. As expected, the IFN response triggered by poly I:C RNA was not dampened by prior incubation of the RNA with ADAR2 (Supplementary Fig. S12). Taken together, this indicates that *in vitro* edited RNAs harbor an editing signature that interferes with MDA5 activation, while random deposition of inosines fails to prevent binding and polymerization of MDA5. As we do not know at which position inosines are replacing guanosines during transcription, the incorporated inosines can be located in base-paired I:C, I:U, or unpaired positions.

It had been reported previously that dsRNA oligos harboring consecutive I:U base pairs can efficiently suppress ISG induction by long dsRNAs. In contrast, the same oligos harboring I:C basepairs fail to do so [50]. We therefore tested whether the induction of ISGs can be suppressed by I:U basepair-containing oligos. Indeed, cotransfection of I:U-pair-containing oligos did suppress the ISG induction by the dsRNAs formed in *Rnf168* or *Zkscan3*. Oligos with I:C basepairs failed to do so (Supplementary Fig. S13).

### *In vitro* editing efficiently prevents stress granule formation by dsRNA substrates

To further compare the impact of inosines introduced by *in vitro* editing with that of inosines introduced by *in vitro* transcription, we transfected cells with an RNA containing the 3′ UTR of *Knl1*. Inosines were introduced either by RNA editing *in vitro* or during *in vitro* transcription. Stress granule formation was monitored by G3BP1 staining. As expected, transfected *Knl1* RNA induced stress granules similar to transfection of poly I:C. Again, *in vitro* editing suppressed stress granule formation, while inosines introduced during *in vitro* transcription failed to do so (Fig. 4B and C). This confirms that inosines introduced by editing where adenosines are converted to inosines are differently sensed by cells than inosines randomly replacing guanosines introduced by *in vitro* transcription.

### *In vitro* editing introduces characteristic, reproducible editing patterns in double-stranded regions

To determine the editing patterns deposited by recombinant ADARs, we reverse transcribed, amplified, and sequenced the *in vitro* edited RNAs. Quantitation of G over A peak heights at editing sites by Sanger sequencing demonstrated that ADARs deposit inosines in a non-stochastic manner, showing editing rates of almost 100% at distinct sites. As only ADAR1p150 can efficiently inhibit MDA5 activation *in vivo*, we were surprised to see that ADAR2 displayed the overall most abundant and strongest editing pattern, while ADAR1p150 and ADAR1p110 were qualitatively and quantitatively less active (Supplementary Fig. S14). However, the extended activity of ADAR2 also fits the observation that *in vitro* editing by any ADAR enzyme can suppress MDA5 activation in cells. It should also be mentioned that editing reactions were carefully titered by increasing time and protein to substrate concentrations. This showed that some sites rapidly reached almost 100% editing with ADAR1p150 or ADAR1p110. Still, even extended incubation times or increased protein concentrations did not lead to increased overall editing at sites that showed medium or low editing levels. ADARs prefer to edit adenosines that are opposite unpaired cytosines [68, 70, 71]. It was therefore interesting to see that A:C base pairs were not always most frequently edited (Supplementary Fig. S15) and those were not always the most highly edited sites (red dots in Supplementary Fig. S14). In Rnf168, for instance, the relative frequency of editing events at base-paired A:U positions was equal to that of A:C positions. Some editing events at A:U basepairs were also highly edited by all three ADARs. In Knl1 and Zkscan3, A:C mismatches were still the relatively most preferred editing sites (Supplementary Fig. S15), although the absolute number of A:U edits obviously exceeds that of editing events at A:C mismatches, which is a simple result of the absolute occurrence of A:U base pairs in dsRNA. Still, we wondered how editing would affect the overall stability of the double-stranded regions. We compared the thermodynamic stability of RNAs with and without inosines at sites showing >25% *in vitro* editing by *in silico* folding (Supplementary Fig. S16). As expected, editing decreased the thermodynamic stability of all RNAs as most editing events were found at A:U base-paired regions. Interestingly, however, the introduction of inosines did also lead to seemingly more stable regions within Zkscan3 (Supplementary Fig. S16). It should be considered, however, that secondary structure prediction for inosine-containing RNAs is based on an incomplete set of parameters [72]. We also tested for the enrichment of unpaired regions in the vicinity of the highly edited adenosines found in *Rnf168, Knl1*, or *Zkscan3*. However, no obvious structural distortion could be observed. Further, the highly edited adenosines were all following the nearest neighbor preferences previously described [68] (Supplementary Fig. S14B).

Given that *in vitro* editing by any of the three ADAR enzymes could efficiently prevent ISG induction and given that ADAR1p150 is the only enzyme that is essentially needed to suppress MDA5 activation *in vivo*, we aimed to compare editing patterns of ADAR2, ADAR1p110, and ADAR1p150 *in vitro* and in cells upon ectopic expression of any of the three ADAR enzymes. To do so, ADAR2, ADAR1p150, or ADAR1p110 were introduced into immortalized murine embryonic fibroblasts (MEFs) derived from completely editing-deficient mice (*Adar1^−/−^; Adarb1^−/−^; Mavs^−/−^*) [23]. Subsequently, RNA was extracted, cDNA was generated, and the 3′ UTRs of endogenous *Rnf168, Knl1*, and *Zkscan3* were amplified. Editing levels were compared by Sanger sequencing (Supplementary Fig. S17). Interestingly, ADAR2 exhibited significantly lower activity in cells than *in vitro*, while the overlap between sites edited by the different ADAR isoforms seemed larger in cells than *in vitro*. This finding is consistent with earlier studies that demonstrated a large overlap between ADAR1 and ADAR2 editing patterns in the mouse [23, 73]. It was also interesting to note that also in cells Rnf168 showed lower levels of editing at A:C mismatches, which normally should be the preferred editing target of all ADARs [68, 70, 71] (indicated by red dots in Supplementary Fig. S17).

**Figure 5.**
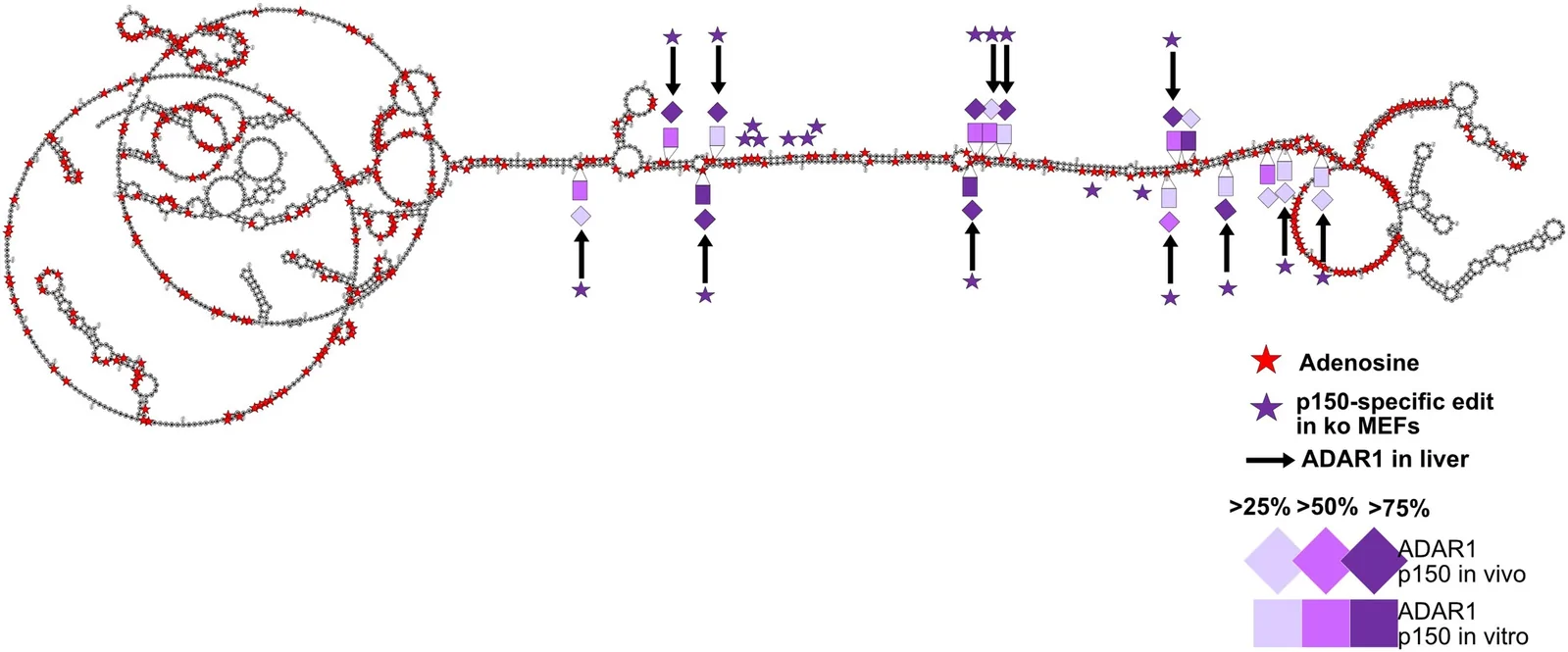
ADAR1p150 shows a distinct editing pattern *in vitro*, in cells, and *in vivo*. Rnf168 was *in vitro* transcribed and edited with recombinant ADAR1p150 (violet squares). Editing of the same endogenous RNA fragment was determined in ADAR-less MEFs that were transduced with a lentiviral vector expressing ADAR1p150. The RNA was extracted, reverse transcribed, and editing levels were determined by Sanger sequencing. Editing levels are indicated and marked (violet diamonds). Also, editing patterns in liver RNA from MAVS-deficient and ADAR1 & MAVS-deficient mice were compared, and ADAR1-specific edits are highlighted by black arrows. Lastly, RNA was extracted from ADAR1p150-specific knockout and wild-type cells treated with interferon-alpha to induce ADAR1 expression. Sequencing of RNAs from these cells enabled the identification of ADAR1p150-specific editing sites within the 3′ UTR of endogenous Rnf168 (violet asterisks). Red asterisks highlight all adenosine residues in the sequence. The p150-specific editing sites showed a high degree of overlap across *in vitro, in cellulo, in vivo* (liver) editing patterns.

Since RNAs edited *in vitro* by ADAR1 and in particular ADAR1p150 are sufficient and essential to suppress interferon signaling in cells, we specifically compared sites edited by ADAR1p150 *in vitro* and in ADAR-less MEFs that express ADAR1p150 from a lentiviral vector [12, 29]. Additionally, we compared these editing patterns with those found to be edited in mouse liver by ADAR1 [59, 74]. Interestingly, the positions edited by ADAR1p150 *in vitro*, in overexpressing cells, and in tissues showed a remarkably high overlap (Fig. 5 and Supplementary Fig. S18). This indicates that ADAR1p150 has a clear preference for specific adenosines or shows a characteristic spacing along dsRNAs, as suggested by others [75, 76]. Recently, an ADAR1p150-specific knockout was published that shows unperturbed expression of ADAR1p110 [12]. We obtained RNAs from cells derived from this knockout and the corresponding wild-type cells. Both wild-type and ADAR1-p150-specific knockout cells were treated with interferon beta to boost ADAR1 expression. Sequencing of the RNAs from these knockout- and wild-type cells allowed us to determine ADAR1p150-specific editing in the 3′ UTRs of endogenous *Rnf168*. Interestingly, the p150-specific editing sites showed a high overlap with the *in vitro, in cellulo*, and in-organ (liver) editing patterns. The intersection of all ADAR1 and ADAR1p150 editing sites highlights that ∼6%–8% of all adenosines in Rnf168, Knl1, and Zkscan168 are edited by ADAR1p150 (Supplementary Fig. S19). This suggests that a selected set of sites is seemingly sufficient to suppress MDA5 activation in cells (Fig. 5).

Taken together, our study demonstrates that RNA editing introduces inosines in a specific and reproducible pattern and that this pattern prevents efficient MDA5 polymerization *in vitro* and *in vivo*. Moreover, we show that the random introduction of inosines by *in vitro* transcription, even if at higher average levels than those introduced by RNA-editing enzymes, is not sufficient to suppress MDA5 activation. Since inosines introduced by *in vitro* transcription substitute G:C or G:U base pairs with I:C or I:U base pairs while inosines introduced by RNA editing largely substitute A:U base pairs with I:U base pairs, it seems that the switch from canonical Watson–Crick base pairing to non-canonical wobble base pairs may be critically required to prevent MDA5 activation.

## Discussion

In our search for critical targets of ADAR1p150 that can activate MDA5 in an unedited state, we modified an RNase-protection assay previously used by the Hur lab [54]. To increase the selectivity of the assay, we used RNAs isolated from MAVS-deficient (*Mavs^−/−^*) and ADAR1 & MAVS-deficient (*Adar1^−/−^; Mavs^−/−^*) mice and selectively focused on RNAs that were protected by MDA5 ^ΔCARD^ in the absence of ADAR1. Protected peaks were mainly in the region of repeats, like SINEs and LINEs in the 3′- and 5′-UTRs of mRNAs, but also in the intronic region of RNAs. While this may seem surprising at first sight, it should be considered that our protection assay was performed on total RNAs, which also contains the nuclear fraction. Given that intronic RNAs are normally retained in the nucleus, such intronic RNAs should not get the chance to activate MDA5, except in the case of aberrant splicing as observed in some tumors and upon splicing inhibition [57, 58, 77–79]. Interestingly, however, such retained intronic sequences predominantly activate the PKR and RNaseL response rather than MDA5 [79]. In any case, we did not further focus on intronic sequences as major sources for MDA5 activation in the absence of ADAR1.

The abundance of LINE elements in the MDA5 ^ΔCARD^ protected fraction was quite surprising (Fig. 1). To this point, it is unclear why such a large fraction of these elements was protected by MDA5 ^ΔCARD^. It might be that, upon isolation and extraction of total RNAs, also intronic LINE elements start hybridizing, therefore generating double-stranded regions that then become exposed and protected by MDA5 ^ΔCARD^. While we cannot exclude that some LINEs base pair and locate to the cytoplasm, their low editing index makes this a rather unlikely scenario (Supplementary Fig. S5). We also tested whether LINEs would support MDA5 ^ΔCARD^-mediated ATP hydrolysis or filament formation (Fig. 2). Here, LINEs seemingly failed to activate MDA5 ^ΔCARD^. Also structurally, LINEs did not form extended double-stranded structures nor were they found in inverted orientation within mRNAs (Supplementary Fig. S6). It therefore seems that MDA5 can bind and protect LINEs *in vitro* but possibly cannot form filaments along them. Nonetheless, we also cannot rule out that MDA5 ^ΔCARD^ used here for RNAse protection assays and subsequent MDA5-mediated activation assays will behave differently from full-length RNA, especially since it has been shown that the CARD domains contribute to MDA5-polymerization. However, given that the RNAs tested here behaved the same way *in vitro* and *in vivo*, we believe that the contribution of the CARD domains will not critically affect the distinction of RNAs with and without inosines.

Our assays clearly showed that the inverted repeats of LTR elements as well as inverted SINEs can activate MDA5 ^ΔCARD^-mediated ATP hydrolysis and filament formation *in vitro*, and further that these elements can also elicit an interferon response and stress granule formation in cells. Most interestingly, our study shows that introduction of inosines at a few distinct adenosine sites can interfere with MDA5 activation while the stochastic replacement of guanosines by inosines fails to do so. While the ability of (almost) perfect dsRNAs to activate MDA5 and downstream signaling was known from several previous publications, it was not known whether the presence of inosines *per se* or the specific introduction of inosines was critically required to suppress MDA5 activation [11, 53, 54]. Here, we have shown that editing by any active ADAR enzyme is sufficient to suppress MDA5 activation. This was surprising, since ADAR1p150 seems critically required for RNA editing *in vivo*, while ADAR2 or ADAR1p110 are not required to prevent MDA5 activation [12]. *In vivo*, the expression patterns of ADAR2, ADAR1p110, and ADAR1p150 differ quite strongly. While ADAR2 is mainly expressed in the nervous system, the vasculature, and the large intestine, ADAR1 is ubiquitously expressed, and ADAR1p150 is expressed under interferon stimuli [80–82]. Thus, loss of ADARp150 may not be compensated by ADAR2 or ADARp110 by failure to suppress MDA5 activation in all tissues. Moreover, only ADAR1p150 localizes to the cytoplasm at significant levels and therefore it seems that only ADAR1p150 can edit cytoplasmically localized double-stranded regions at significant levels.

When we compared the overlapping editing patterns introduced by ADAR1p150 *in vitro* and in cells overexpressing ADAR1p150 with the editing patterns found to be specific for ADAR1 in liver RNA as well as ADAR1p150-specific editing patterns deduced from sequencing specific transcripts of ADAR1p150 knockout cells, a surprisingly small number of highly edited sites remains (Fig. 5). This suggests that a small number of editing events that occur at high frequency may be sufficient to suppress MDA5 activation. Interestingly, many editing events affect A:U basepairs. In fact, A:U basepairs are also most abundant in the dsRNA structures studied here. In relative amounts, however, A:C mismatched basepairs were still the preferred editing sites for all ADAR enzymes in Knl1 and Zkscan3 [68, 70, 71]. Editing in Rnf168, in contrast, was found at same frequencies in A:U base-paired regions as in A:C mismatched regions (Supplementary Fig. S15). Given the high number of I:U base pairs introduced by editing and the fact that inosines introduced by transcription did not affect MDA5 activation, it suggests that I:U wobble base pairs may be critically involved in preventing MDA5 polymerization. I:U base pairs are predicted to mimic G:U wobble base pairs. However, a recent analysis of helices containing multiple I:U base pairs demonstrated an expansion of the helix accompanied by strong backbone distortion. Thus, I:U base pairs may affect helical geometry more strongly than previously anticipated, a finding also supported by recent structural studies [66].

Editing of adenosines can occur across uracils and cytosines and, to a much lower extent, across adenosines and guanosines [68, 71]. Inosines introduced by in vitro transcription could, in principle, occur across any nucleotide, depending on the folding of the final RNA. As I:U base pairs introduce wobble base pairs and lead to the backbone distortion possibly recognized by MDA5 [66], we counted the number of I:U, I:C, and I:G/A base pairs introduced by RNA editing versus those theoretically introduced during in vitro transcription. (As we cannot detect the position of inosine incorporation during transcription, we considered any guanosine within a dsRNA region to be a putative inosine-containing spot). As expected, the majority of edits ∼85% lead to I:U wobble base pairs. In contrast, inosines introduced by *in vitro* transcription only led to a minor extent ∼8%–11% to I:U wobble base pairs (Supplementary Fig. S20). However, structure mapping of RNAs will be required to address this point in more detail. Structural studies have indicated that MDA5 can assess the quality of base pairing in dsRNAs [3, 61]. This would be in line with our findings that suggest that only the change from a Watson–Crick A:U base pair to an I:U wobble base pair can interfere with MDA5 activation, while a G:C base pair into an isosteric I:C base pair, as generated by the replacement of guanosines by inosines during transcription, would fail to do so. Assuming that only a small number of specific inosines is required to prevent MDA5 activation by our model substrates, it will be interesting to synthesize such model substrates with a minimal number of inosines and to test those for their impact on MDA5 activation in cells.

It is also surprising to see that *in vitro* editing efficiently prevents all filament formation, given that some sites are only edited to 70% or even less. This makes it likely that some molecules evade editing altogether. Therefore, it might be that a sufficient number of edited RNAs can inactivate MDA5, thereby preventing its activation by a small number of unedited molecules. Moreover, the study performed here only considers MDA5 in the absence of its CARD domains and also in the absence of its partner LGP2 [83, 84]. Clearly, in cells and in combination with other proteins, editing may affect MDA5 polymerization to different extents. Also, as the structure and shape of RNA in the presence of inosine wobble base pairs are not fully understood, further structural studies will be required to understand how inosines can prevent MDA5 polymerization.

## Supplementary Material

gkag845_Supplemental_Files

## Data Availability

Sequencing data has been deposited at GEO and is available under accession number GSE293616. Editing analysis of protected dsRNA is available in GitHub, https://github.com/BioinfoUNIBA/Mouse-dsRNA-mda5, and Zenodo, https://doi.org/10.5281/zenodo.17876716.
