# Supplementary material for: Distinguishing self from non-self RNA by editing-specific inosine patterns
Source: Nucleic Acids Res. 2026 Aug 24;54(16):gkag845. doi: 10.1093/nar/gkag845 (PMC13501140; doi:10.1093/nar/gkag845)
Supplement: Supplementary file 1 [file gkag845_supplemental_files.zip › Varada_supplementary_information clean.pdf]

Supplementary material to *Varada et al.,*

Distinguishing self from non-self RNA by editing-specific inosine patterns

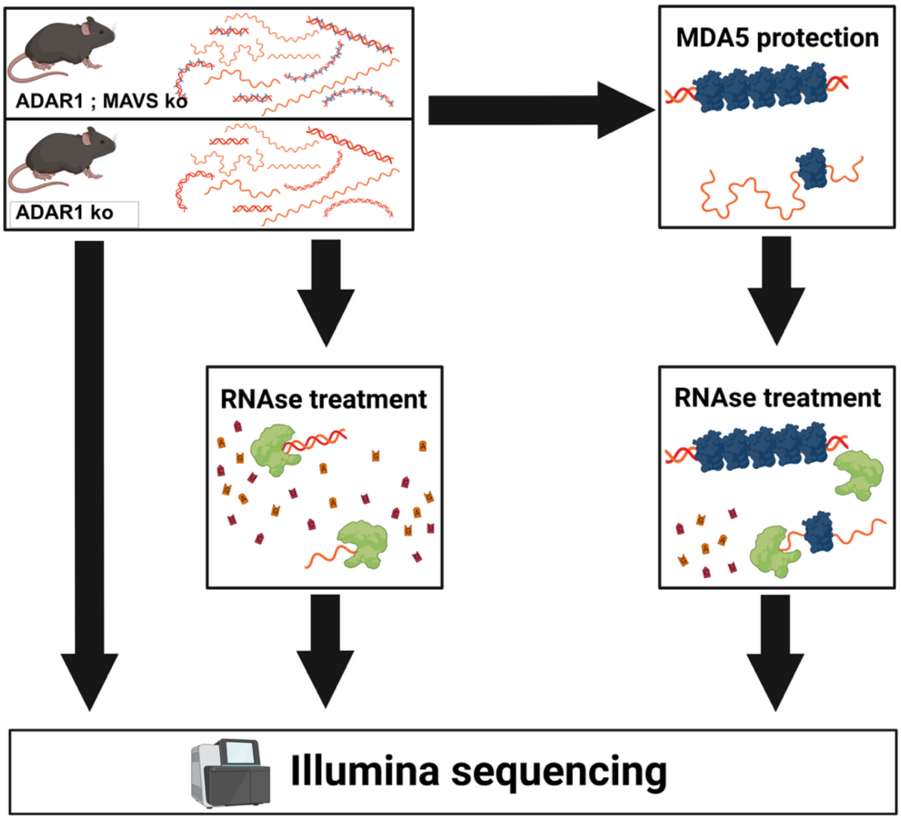

Supplementary figure 1:
Characterization of MDA5 protected RNAs. Total RNA from mouse liver was isolated. An aliquot of the total RNA was used for library preparation to allow quantification of expressed genes (left arrow). An aliquot of the total liver RNA was treated with an RNAse mixture to assess RNAse resistant sites (middle arrow). A fraction of the total liver RNA was incubated with recombinant MDA5 and treated with RNAses. The protected RNAs were used for Illumina library preparation (right arrow). Created in BioRender. Rajendra, V. (2025) https://BioRender.com/fheaon5

Supplementary table 1:
Sequencing IDs, origin of RNA, genotype of mice, treatment of samples and corresponding read numbers are indicated.

**ID**	**organ**	**genotype**	**treatment**	**read numbers**
RV01001	liver	*Mavs-/-*	MDA5 protected	17.004.948
RV01004	liver	*Mavs-/-*	MDA5 protected	17.750.618
RV01007	liver	*Mavs-/-*	MDA5 protected	18.850.273
RV01002	liver	*Mavs-/-*	RNAse only	10.283.844
RV01005	liver	*Mavs-/-*	RNAse only	9.105.496
RV01008	liver	*Mavs-/-*	RNAse only	14.786.880
RV01003	liver	*Mavs-/-*	input	28.241.330
RV01006	liver	*Mavs-/-*	input	29.169.825
RV01009	liver	*Mavs-/-*	input	30.849.504
RV01010	liver	*Adar-/-; Mavs-/-*	MDA5 protected	17.137.732
RV01013	liver	*Adar-/-; Mavs-/-*	MDA5 protected	17.662.011
RV01016	liver	*Adar-/-; Mavs-/-*	MDA5 protected	18.872.072
RV01011	liver	*Adar-/-; Mavs-/-*	RNAse only	12.369.156
RV01014	liver	*Adar-/-; Mavs-/-*	RNAse only	10.022.532
RV01017	liver	*Adar-/-; Mavs-/-*	RNAse only	9.301.382
RV01012	liver	*Adar-/-; Mavs-/-*	input	131.278.150
RV01015	liver	*Adar-/-; Mavs-/-*	input	31.623.717
RV01018	liver	*Adar-/-; Mavs-/-*	input	32.712.242

Supplementary table 2:

List of primers used

**Oligos for:**	**Oligo name**	**Target**	**Sequence(5`->3`)**
**In-vitro transcription templates cloning** (All Forward primers have Sac1 and HindIII sites, and reverse primers have Kp1 and Sall sites for cloning). LINE-b primers have the XhoI site	Rnf168 FP	Rnf168 3`UTR	ATATGAGCTCAAGCTTCCAAGCAAAGCATGGGGAAAGAGTTCTATAATCCAG
	Rnf168 RP		GCATATGTCGACGGTACCTGCATTAGGACAGTTTATTATGTTCACAGG
	Knl1 FP	Knl1 3`UTR	ATATGAGCTCAAGCTTAAGTTTTCCCGTAAGACCCACAGATCACAGC
	Knl RP		GCATATGTCGACGGTACCGCCTGTAAAGCATTCTTTATAGATAAGGAAATGACC
	Zkscan3 fragment1 FP	Zkscan3 3`UTR	ATATGAGCTCAAGCTTGCTACAACCCTTCCTTTCTTATTC
	Zkscan3 fragment1 RP		ATATGTCGACGGTACCCAAGCCCTTCTGGAGGATCCC
	Zkscan3 fragment2 FP		ATATGAGCTCAAGCTTGCTTGCTCAGCTGGCTCAGCC
	Zkscan3 fragment2 RP		ATATGTCGACGGTACCTCTTCCATTTGGTAGGTCTGTGGC
	LINE-b FP	Lx3B	CCACATCTCGAGTAGAGGGCTAATATCCAAAATATATG
	LINE-b RP		TTACACTCGAGTCTTCATCTCCCTCCCAGTC
**Editing detecting primers**	Rnf168 Fragment 1 FP	Rnf168 3`UTR	CCAAGCAAAGCATGGGGAAAGAG
	Rnf168 Fragment 1 RP		AAAGTTTTGGAAAGGGAAGTGGCA
	Rnf168 Fragment 2 FP		TGCCACTTCCCTTTCCAAAACTTT
	Rnf168 Fragment 2 RP		CAAAGGAAATGCTAAAATGCTTAGA
	Rnf168 Fragment 3 FP		TTAAATGAATGGGTAAGGAGTCCA
	Rnf168 Fragment 3 RP		CTGTAACTAATTGATGTTGATACTT
	Rnf168 Fragment 4 FP		GACAGCTACAGTGTACTTACATATAAT
	Rnf168 Fragment 4 RP		TTTTTGAGGCAGGGTTTCTCTGTATAG
	Rnf168 Fragment 5 FP		TTATTATACATAAGTACACTGTAGC
	Rnf168 Fragment 5 RP		GATTAAAGAGAATGTTCTGCTACA
	Rnf168 Fragment 6 FP		GGAACTGAATGGGTAATCAGAAACT
	Rnf168 Fragment 6 RP		TGCATTAGGACAGTTTATTATGTTCAC
**Editing detecting primers**	Knl1 fragment 1 FP	Knl1 3` UTR	AAGTTTTCCCGTAAGACCCAC
	Knl1 fragment 1 RP		CCCAAGTGCTGGGATTAAAGG
	Knl1 fragment 2 FP		CTTAACATCATATAAAAGTAGCA
	Knl1 fragment 2 RP		CTAATGATACAAATTAATTATGATATC
	Knl1 fragment 3 FP		CTATACAGAGAAACCCTGTCTTG
	Knl1 fragment 3 RP		CAAATAAAAACAACACCAAACACC
	Knl1 fragment 4 FP		GAGGTAGTGCACAAAGATTAAG
	Knl1 fragment 4 RP		GTGTTTCCACTCTAGACATATCT
	Knl1 fragment 5 FP		CACTGCCTAGCTAATCATTTCTTG
	Knl1 fragment 5 RP		ATGGCAACCATTCCAAAAGTTG
	Knl1 fragment 6 FP		ACACCAACAGAACATATCTATT
	Knl1 fragment 6 RP		GCCTGTAAAGCATTCTTTATAG-
**Editing detecting primers**	Zkscan3 fragment 1 FP	Zkscan3 3`UTR	GCTACAACCCTTCCTTTCTTATTCATTCCC
	Zkscan3 fragment 1 RP		AGTATGTCACTGTGGGTGTGAGCTTTG
	Zkscan3 fragment 2 FP		GAAGGCTGCTAGGAGAAGACTGGCTCCC
	Zkscan3 fragment 2 RP		GTGGTTGCTGGGAATTGAACTCAGGACC
	Zkscan3 fragment 3 FP		AGAGAGATAGATGGCTCAGCAGTTA
	Zkscan3 fragment 3 RP		GTCCTGGCACTCACTTTGTAGACC
	Zkscan3 fragment 4 FP		GAGGCAGAGGCAGGCGGATTTCTG
	Zkscan3 fragment 4 RP		GAGGATCCCAGAAGACTGAGTAAATCCC
	Zkscan3 fragment 5 FP		CCAGGCAAAACCAAACCAGCAGTA
	Zkscan3 fragment 5 RP		TAAAGATTGTATTGGTTGGTTTTGTGGC
	Zkscan3 fragment 6 FP		ATTTCCTTAACTGAGGCTGTTTCCTCTC
	Zkscan3 fragment 6 RP		CTGTGCCCTCAGGTTCCTGTATGAGTT
	Zkscan3 fragment 7 FP		AGGGTTTATTTTTATCCCATAGTCTTTG
	Zkscan3 fragment 7 RP		CTGGTCAGGTAGTCCTGATTGTAG
	Zkscan3 fragment 8 FP		CTGCCTGAATGCTGCCATGTTCCT
	Zkscan3 fragment 8 RP		TCTTCCATTTGGTAGGTCTGTGGCAAGTC
**Editing detecting primers**	Lx3B fragment1 FP	Lx3B	ATTGGGAATAAAATTTTACTAACCCc
	Lx3B fragment1 RP		CATTCTTGCCAACATGTTCTGTC
	Lx3B fragment2 FP		GAGATTTGACCTTATACCACTCAG
	Lx3B fragment2 RP		TTGTTTCCAGTTTCCAGCTATC
	Lx3B fragment3 FP		AGGTGTCCCACTATGTTCATAG
	Lx3B fragment3 RP		TTCTTCTCCCTTAAAAGTGGAATTGAG
	Lx3B fragment4 FP		CTCAGGATATAATCCACAGATCT
	Lx3B fragment4 RP		CTACTAGAGGTGGACTCTACAA
	Lx3B fragment5 FP		GGAGGGACCTTAGATAGAATC
	Lx3B fragment5 RP		CCTCCTTAGCTTCTTCTAGC
	Lx3B fragment6 FP		GATGGACAGAAGCTGGTAATC
	Lx3B fragment6 RP		TTTTTTTACACTCCAGTCTTCATC
**Editing detecting primers**	ZNF-708 fragment1 FP	ZNF-708 3`UTR	AGCTTGTGGAAATATCTAAGG
	ZNF-708 fragment1 RP		CCAAGTAGCTGAGACTACA
	ZNF-708 fragment2 FP		GGTAAAACCCTGTCTCTACT
	ZNF-708 fragment2 RP		CCTTTCTAGTTGAAGCATCC
	ZNF-708 fragment3 FP		CAGAGTGAGACTCCATCT
	ZNF-708 fragment3 RP		TGAATGTAATTACAAACTTCC
	ZNF-708 fragment4 FP		CCCAAACTAACGGTAGATAAA
	ZNF-708 fragment4 RP		CCTGTAATCTCAGCTACTC
	ZNF-708 fragment5 FP		CACAATCTCAGCTCGCTG
	ZNF-708 fragment5 RP		GTACCGAATTCAAAGAACAC
	ZNF-708 fragment6 FP		GCGGTGATGCCTTTGAGGG
	ZNF-708 fragment6 RP		CCGTTCGGAGGCCGACGG
**Editing detecting primers**	AdML Fragment 1 FP	AdML	TAATACGACTCACTATAGGGCT
	AdML Fragment 1 RP		AGATTGTCTTTTCTGACCAGA
	AdML Fragment 2 FP		GCGGTGATGCCTTTGAGGG
	AdML Fragment 2 RP		CCGTTCGGAGGCCGACGG
**ISG Primers qPCR**	Actin-B FP	Actin-B	CTTTGCAGCTCCTTCGTTGC
	Actin-B RP		ACGATGGAGGGGAATACAGC
	Stat1 FP	Stat1	GCTGCCTATGATgTCTCGTTT
	Stat1 RP		TGG AAAATGCCCA AAGATTT
	Irf7 FP	Irf7	GACCCTCAACACCCTAATACCT
	Irf7 RP		CATTGCTCCGTCTACACAGG
	Ifit1 FP	Ifit1	TGCCAATGAATGAACCAGCA
	Ifit1 RP		GCAGACTGTCACACCAACTG
	Gapdh FP	Gapdh	TTGAGGTCAATGAAGGGGTC
	Gapdh RP		TCGTCCCGTAGACAAAATGG
cotransfection of IU oligos	sense	control	GGUCCGGC**UCCC**CCAAAUGdTdT
	anti		CAUUUGGGGGAGCCGGACCdTdT
	sense	cIU	GGUCCGGC**IIUI**CCAAAUGdTdT
	anti		CAUUUGGUIUUGCCGGACCdTdT

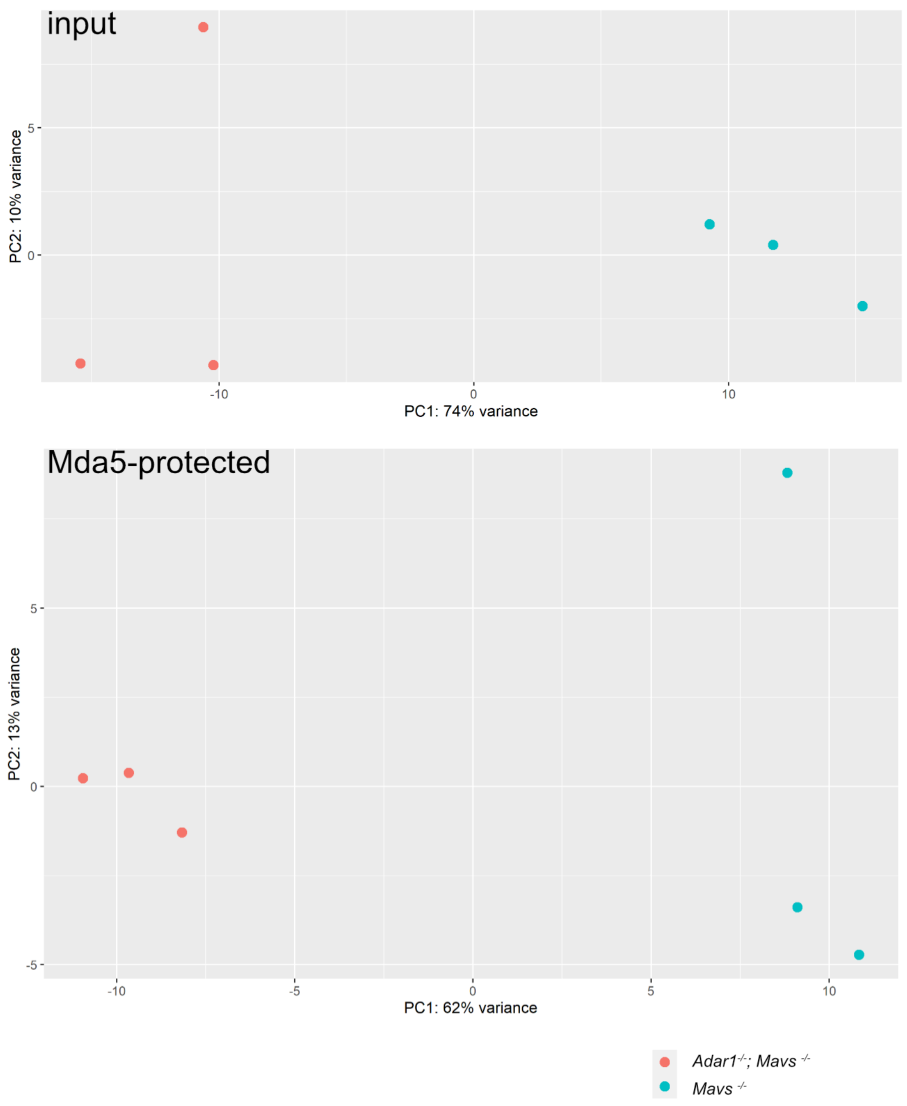

Supplementary figure 2:
Principle component analysis demonstrates a clear separation of input (top) and MDA5-protected (bottom) RNAs isolated from livers of *Adar1*$^{-/-}$*; Mavs*$^{-/-}$ double-knock (red dots) out mice or *Mavs*$^{-/-}$ (turquoise dots) control mice.

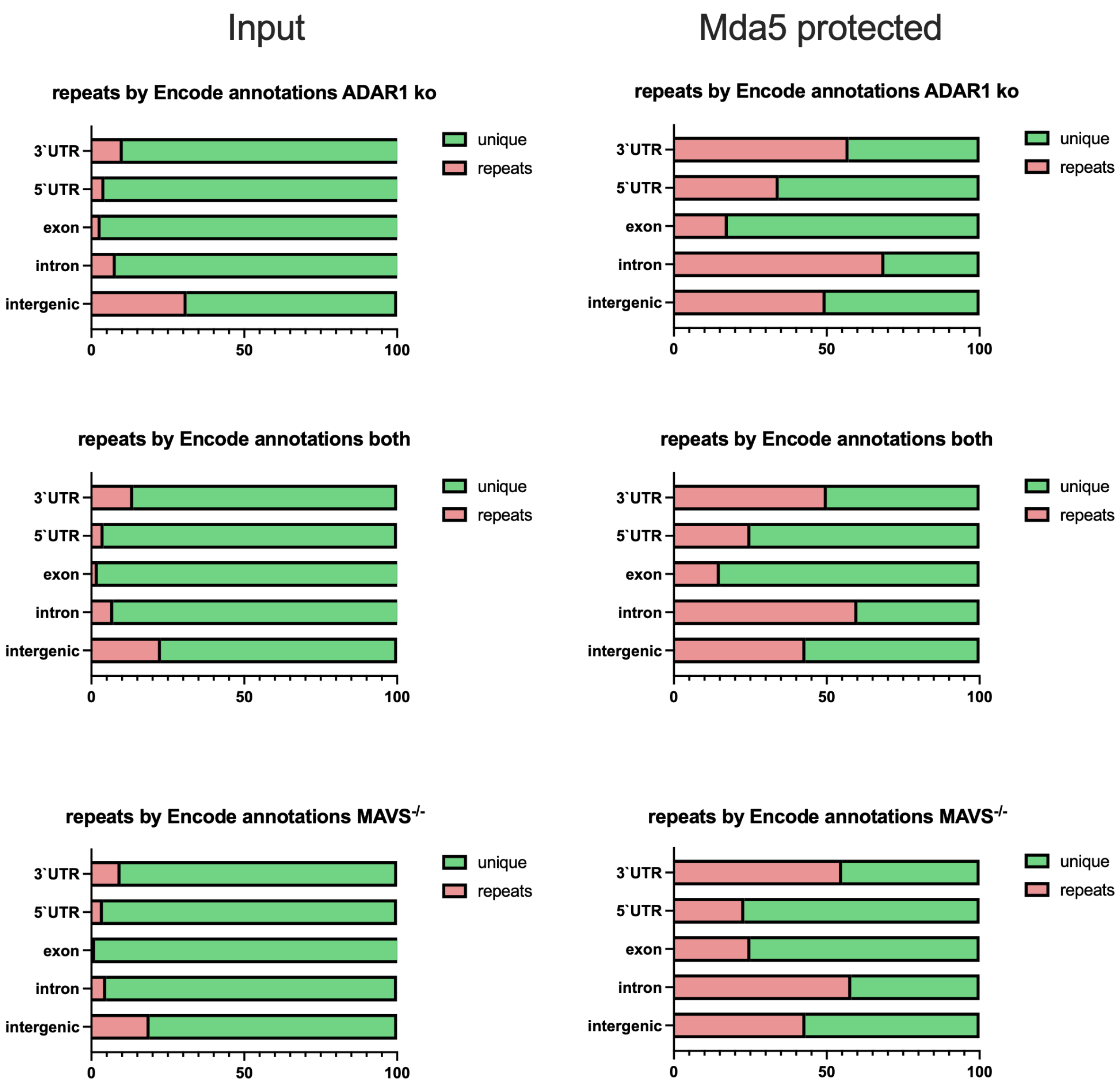

Supplementary figure 3: Fraction of repeat-derived reads in input and MDA5-protected samples as found in MAVS knockout (*Mavs*$^{-/-}$) or ADAR1 & MAVS knockout (*Adar*$^{-/-}$, *Mavs*$^{-/-}$) or in reads of both genotypes. In the MDA5-protected samples, the largest proportion of repeats is found in introns, 3’UTRs and intergenic regions.

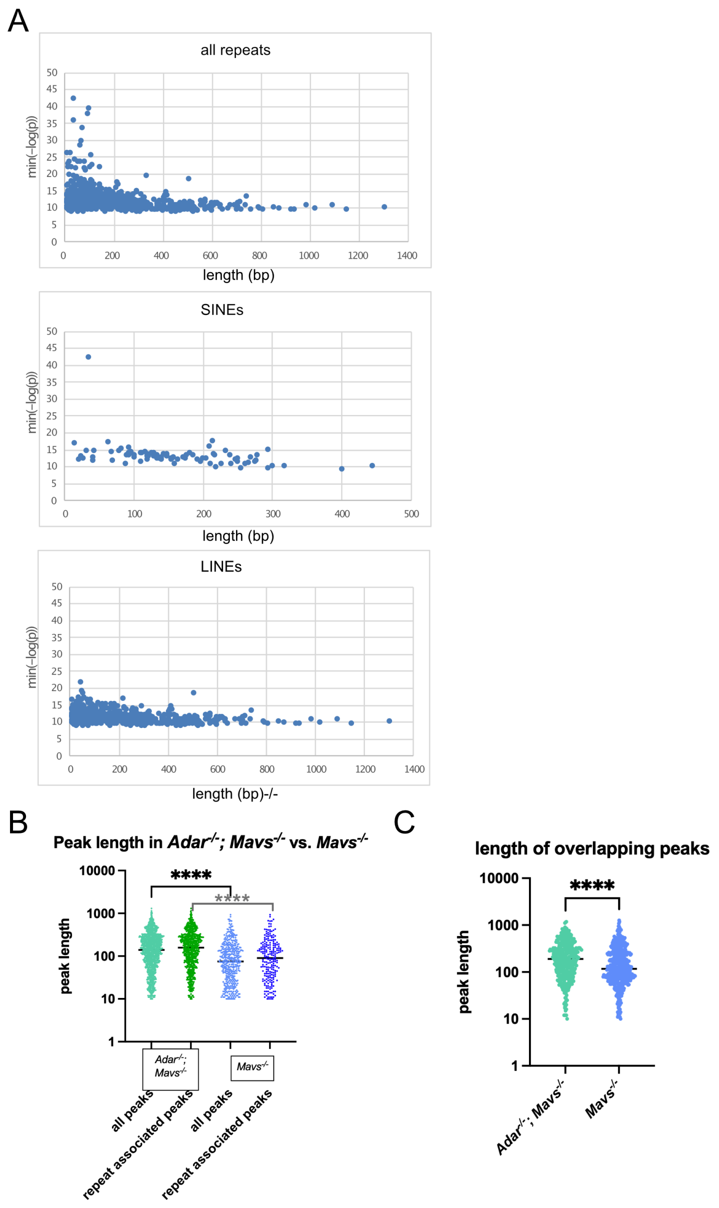

Supplementary Figure 4: A) Length distribution of fragments and significance of protection by MDA5 in *Adar*$^{-/-}$*; Mavs*$^{-/-}$ mice. Protected peaks can be as long as 1300 nt in length. Peaks derived from SINE elements are naturally shorter than those derived from LINEs. Interestingly, the most significantly protected peaks had lengths between 50 and 100 nt. **B)** average protected peak lengths are longer in *Adar*$^{-/-}$*;Mavs*$^{-/-}$ individuals than in *Mavs*$^{-/-}$ samples. Scatter plot and median of peak lengths are indicated. MDA5 protected peaks from wild-type and double mutant individuals are given for all peaks and repeat-containing peaks.Differences between genotypes are highly significant **** p<0.0001. **C)** scatter plots and median of peak lengths for peaks that are found in both *Adar*$^{-/-}$*;Mavs*$^{-/-}$and *Mavs*$^{-/-}$ mice.

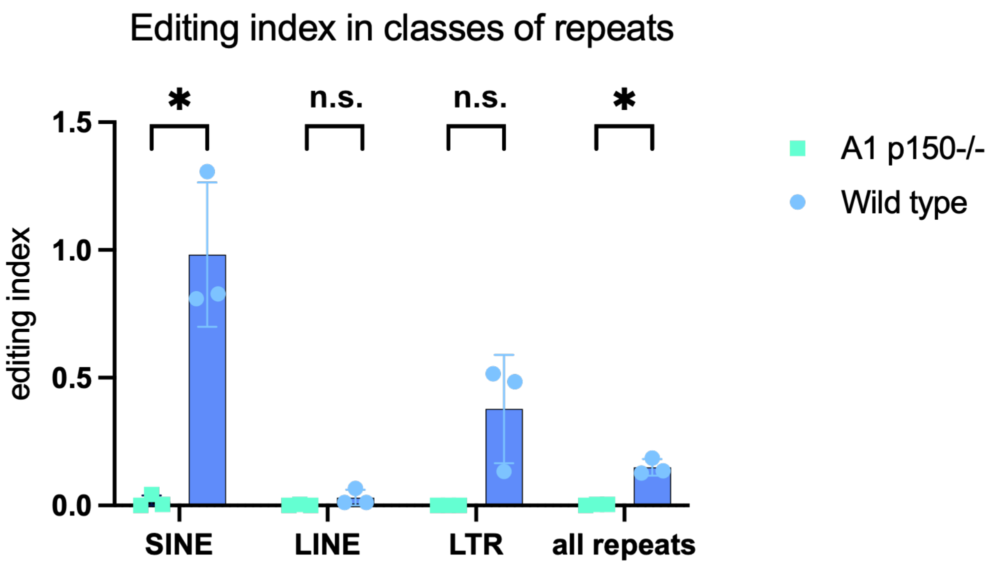

Supplementary figure 5: Editing index across classes of repeats in the absence and presence of ADAR1p150. The editing index was calculated for SINEs, LINEs, LTR elements, and all repeat-associated sequences from MEFs derived from wild type mice (round symbols, and mice only deficient in ADAR1p150 [1]. High editing levels were observed for SINEs and LTRs while LINEs were almost not edited. However, only SINEs showed a reduction in editing upon depletion of ADAR1p150. (SINE p=0.0267, LINE p= 0.256, LTR p=0.091, all repeats p= 0.015). Asterisks denote p-values <0.05.

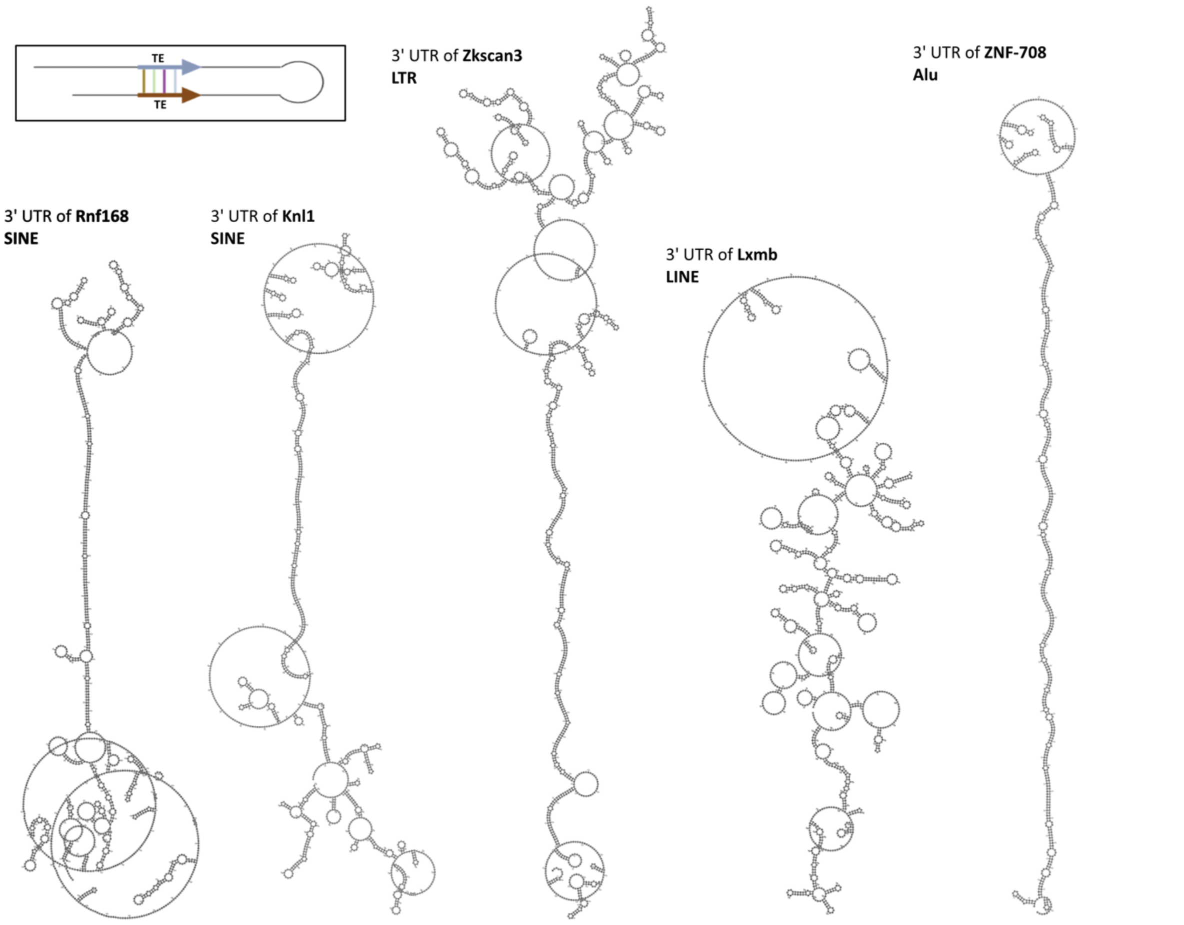

Supplementary figure 6: Secondary structure prediction of MDA5-protected RNA fragments

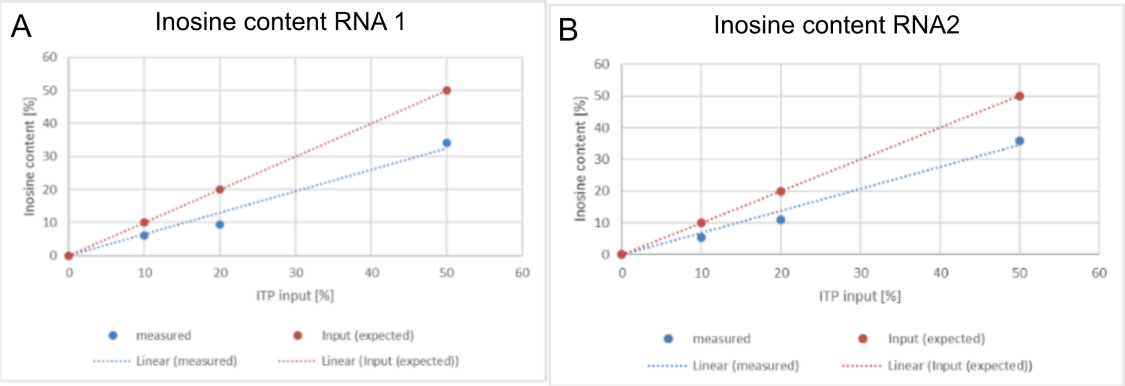

C Inosine incorporation by transcription

sample	amount I during IVT	I/(I+G)
1	RNA1, 0% I	0 %
2	RNA1, 10% I	6.03 %
3	RNA1, 20% I	9.28 %
4	RNA1, 50% I	34.17 %
5	RNA2, 0% I	0 %
6	RNA2, 10% I	5.49 %
7	RNA2, 20% I	11.05 %
8	RNA2, 50% I	36.00 %

D Inosine incorporation by in vitro editing

RNA	ADAR2 G/(A+G)	ADAR1p110 G/(A+G)	ADAR1p150 G/(A+G)
Zkscan3	11.3%	8.3%	7.7%
Rnf168	14.0%	10.4%	10.8%
Knl1	7.0%	6.2%	6.8%
ZNF708	26.9%	25.6%	22.9%

Supplementary figure 7:Inosine incorporation by T7 polymerase and by in vitro editing
In vitro transcriptions were set up with 500µM rNTPs where GTP was substitutes with 10, 20, and 50% ITP. Concentrations higher than 50% ITP inhibited transcription. The concentration of incorporated ITP was determined by HPLC of hydrolyzed RNAs **A)** *Rnf168*, **B)** *Knl1*. **C)** shows levels of input inosine and ratios of inosines over the sum of I & G. **D)** Amount of editing measured for *Zkscan3* (LTR), *Rnf168, Knl1* (both SINEs) and *ZNF-708* (human Alu). Editing levels were measured by Sanger sequencing of RT-PCR products where the hight of the G peak was divided by the sum of the heights of the A and G peaks, for all adenosines present in the double-stranded structure.

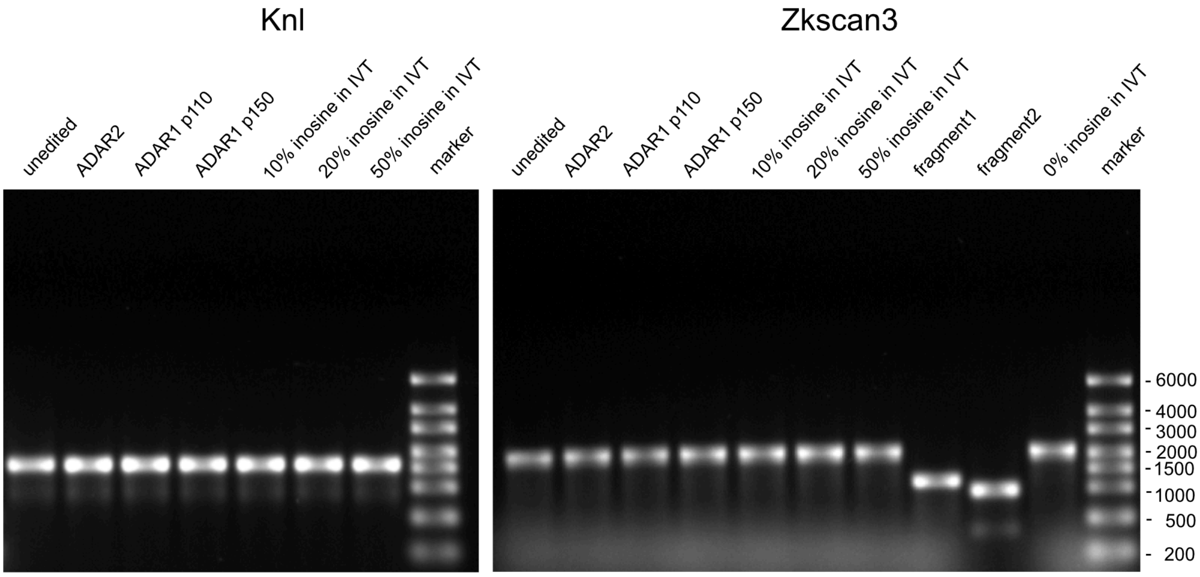

Supplementary figure 8: Control gel of RNAs edited *in vitro* and of RNAs transcribed by partially replacing guanosines with inosines. After *in vitro* editing or *in vitro* transcription in the presence of inosine, RNAs were extracted with phenol, phenol:chloroform, and chloroform:isoamylalcohol. Subsequently, RNAs were precipitated with ethanol, washed with 70% ethanol, air dried, and resuspended in water at 200ng/µl. The equivalent of 200-300ng RNA was loaded on an RNA-formaldehyde gel. The left gel shows the *in vitro* transcribed RNA of Knl either unedited, edited with ADAR2, ADAR1p110 or ADAR1p150. RNAs were also transcribed by replacing GTP with 10%, 20% or 50% inosine during *in vitro* transcription. The gel for Zkscan shows the same treatments and also shows the in vitro transcript of the first half (fragment 1) or the second half (fragment 2) of Zkscan next to an RNA marker. Note that the integrity of the RNA was not affected by the *in vitro* editing reaction.

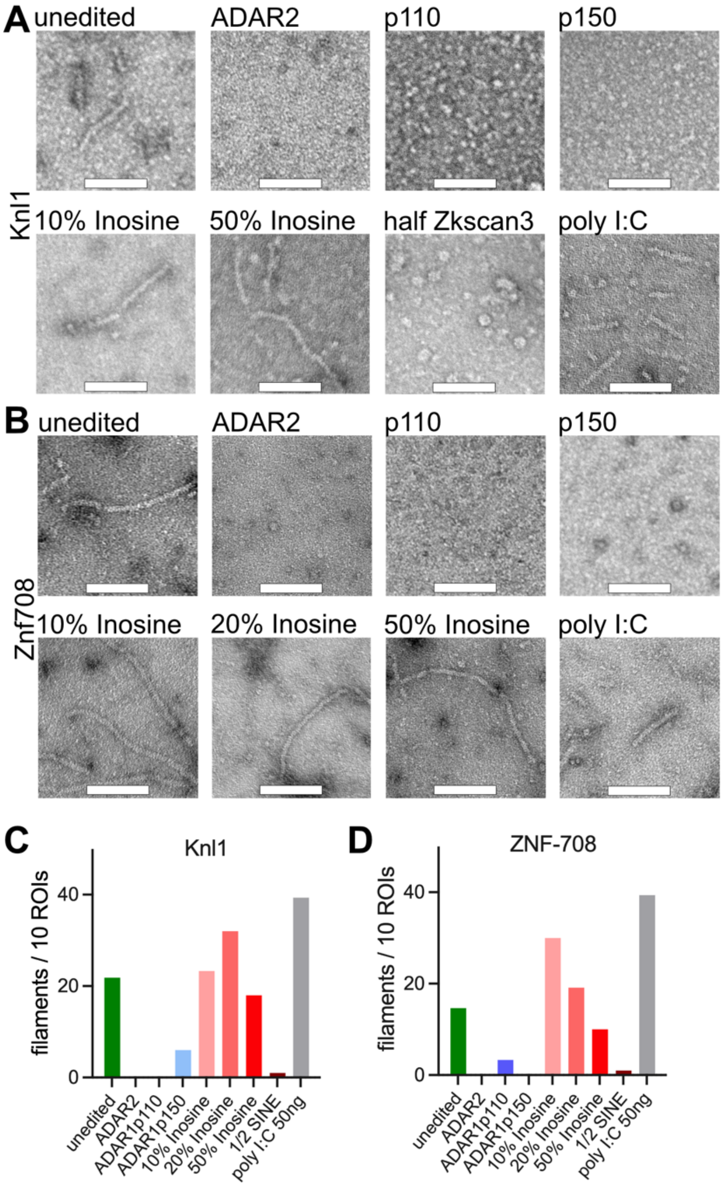

Supplementary figure 9: Only inosines incorporated by in vitro editing can suppress MDA5 filament formation.

A) The 3' UTR of mouse *Knl1* carrying two inverted SINEs was i*n vitro* transcribed in the presence or absence of 10, 20, or 50% inosine triphosphate or in-vitro edited by recombinant ADAR1p110, ADAR1p150 or ADAR2 and incubated with recombinant MDA5 lacking the CARD domains in the presence of non-hydrolyzable ATP. Unedited RNA and RNA with inosines incorporated during in vitro transcription support MDA5 filament formation. In contrast, RNAs that were edited *in vitro* by any ADAR enzyme no longer support filament formation. **B)** The experiment described in A) was performed with *in vitro* transcribed 3' UTR of human Znf708 which carries two inverted Alu elements. Again, only unedited RNA or RNAs carrying inosines incorporated by in-vitro transcription support MDA5 filament formation, while in vitro edited RNAs fail to support MDA5 filaments.

C & D quantitation of 10 ROIs of the data shown in **A** and **B**, respectively.

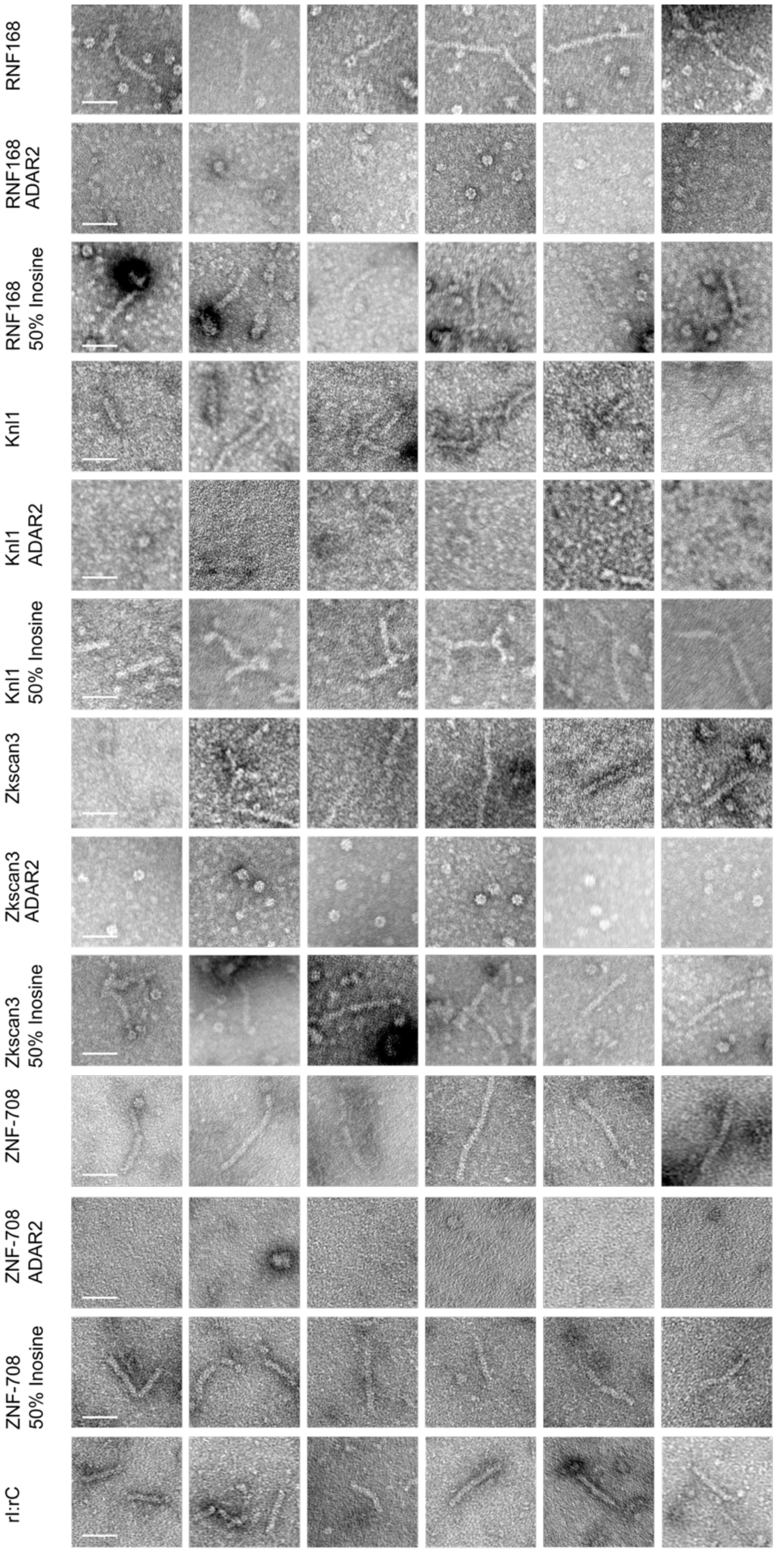

Supplementary Figure 10: Representative images of filaments formed on dsRNAs of Rnf168, Knl1, Zkscan3, the human Alu ZNF-708 amd poly I:C. The mouse B1 elements Rnf 168 and Knl1 support shorter filaments of 50-80 nm while Zkscan supports the formation of longer filaments (80-100nm). The human Alu containing 3’ UTR of can form dsRNA structures of 250 bp and can support filament formation of ~150 nm. Editing by ADAR2 suppresses efficient polymer formation while random replacement of Gs by Is has seemingly no effect on the efficiency of MDA5 filament formation.

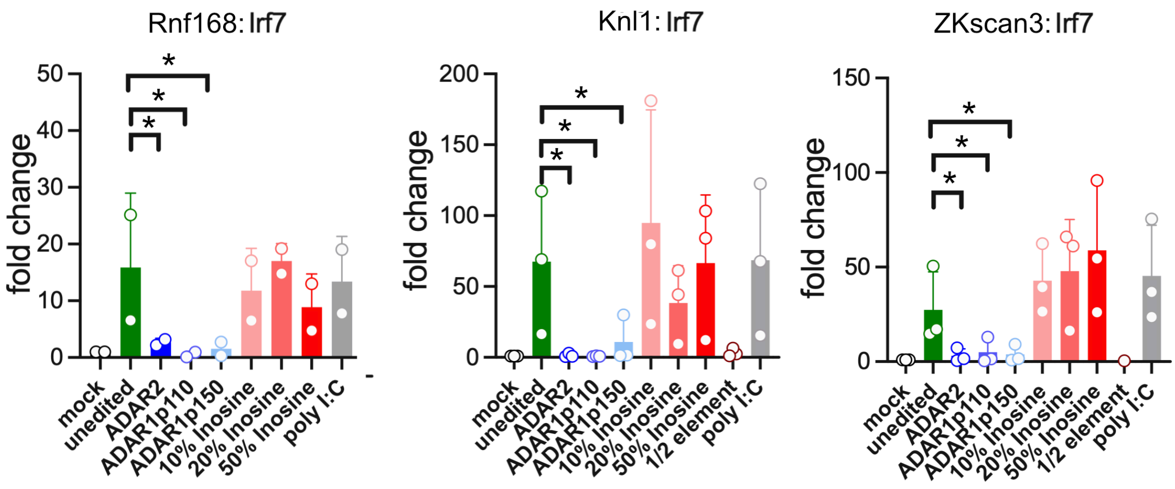

Supplementary figure 11: The double-stranded UTRs of *Rnf168, Knl1* and the LTR element *Zkscan3* induce an interferon response stimulating Irf7 expression that is antagonized by in vitro editing but not by inosines introduced by in vitro transcription.

Immortalized, editing-deficient mouse embryonic fibroblasts were transfected with 300ng of in vitro transcribed and dephosphorylated RNA originating from the 3' UTRs of the mouse *Rnf168* or *Knl1* genes harboring two inverted SINEs. Also the *in vitro* transcribed LTR element located at the Zkscan3 gene was transcribed. The RNA was either transfected as is, or was treated with recombinant ADAR2, ADAR1p110 or ADAR1p150. Alternatively, GTP was substituted by ITP during in vitro transcription to 10%, 20% or 50%, as indicated. 24 hrs post transfection total RNA was extracted, cDNA was synthesized from 200ng RNA and the cDNA was used for qPCR to determine expression levels of *Irf7*. *Gapdh* was used as a reference. the fold change induction over Mock transfected (transfection reagent only) was calculated and is plotted on the Y-axes. In vitro transcribed RNAs or RNAs in which inosines were substituting guanosines during in vitro transcription can boost IRF7 expression, comparable to rI:rC RNA. In contrast, RNAs edited by ADAR2, ADAR1p110 or ADAR1p150 fail to cause an IFN response.

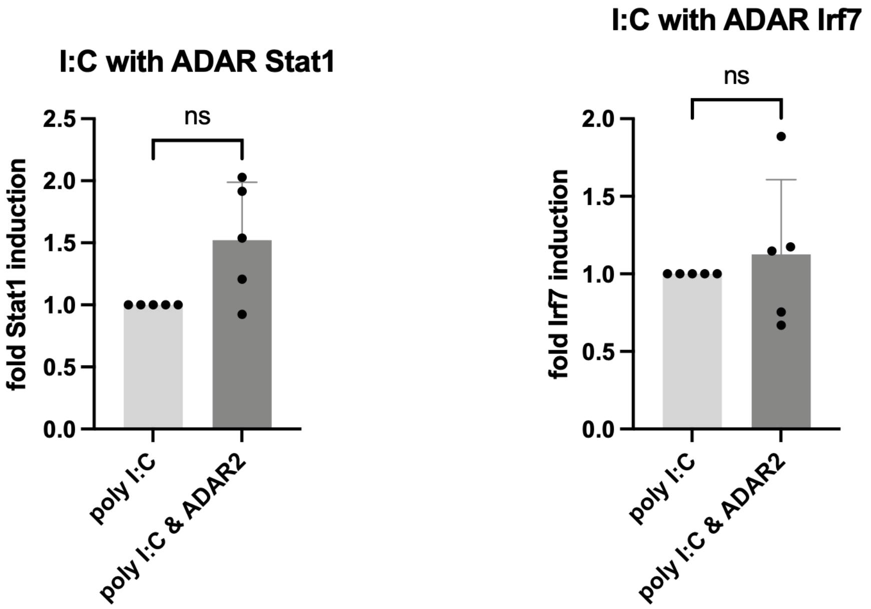

Supplementary figure 12: ADAR2 treatment does not affect induction of ISGs by poly I:C. Poly I:C RNA was incubated with ADAR2 for 30 minutes, followed by phenol extraction, ethanol precipitation and quantification. Equal amounts of untreated or ADAR2-treated poly I:C were transfected into mouse embryonic fibroblasts. After 24hrs total RNA was extracted and qPCR assays for the induction of Stat1 or Irf7 relative to actin were performed. Treatment with ADAR2 did not diminish the induction of ISGs.

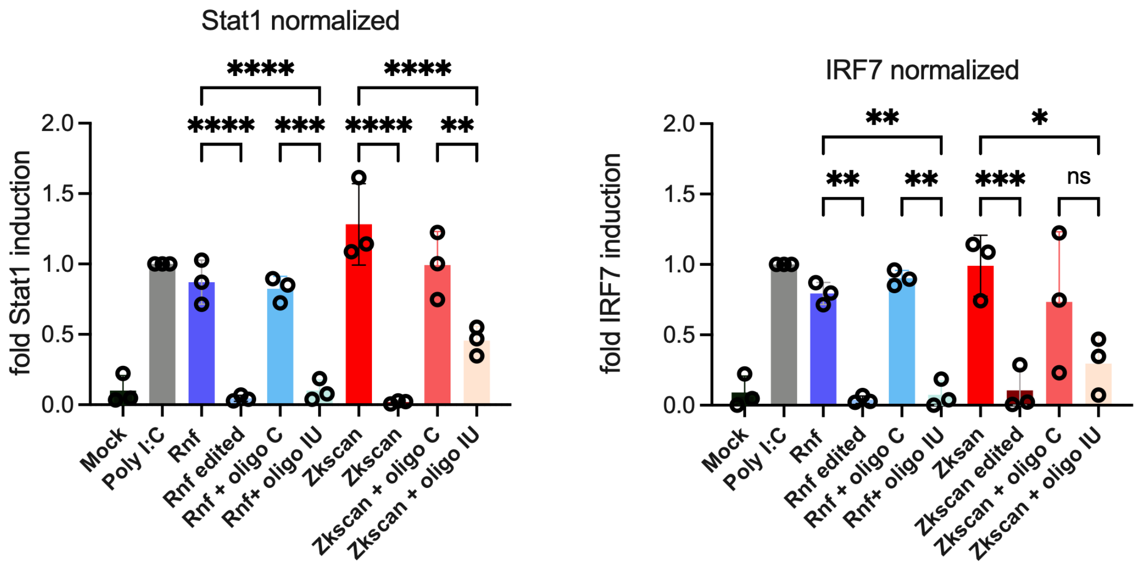

Supplementary figure 13: Oligos harboring I:U basepairs can suppress ISG expression triggered by the dsRNAs found in *Rnf168* or *Zkscan3.* Poly I:C, Rnf RNA, edited Rnf RNA, or RNF RNA together with an IU or control (C) oligo were transfected into MEF cells. After 24hrs ISG induction for Stat1 or Irf7 was measured by qRT-PCR. ISG induction was normalized relative to induction by polyI:C. I:U containing oligos can successfully suppress ISG induction by RNF168 (left) or Zkscan (right)

A

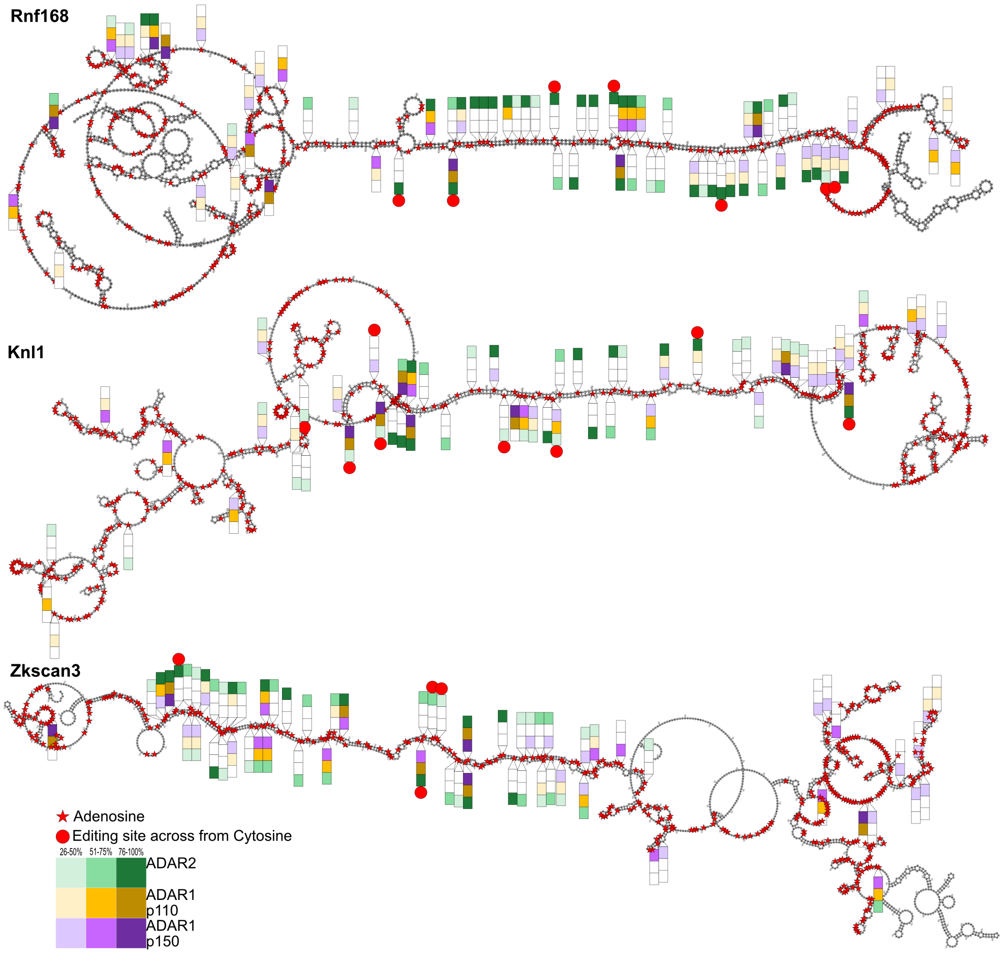

B

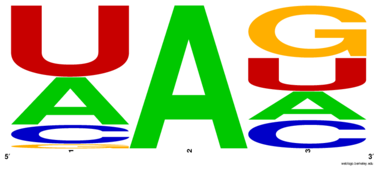

Supplementary Figure 14: ***In vitro* editing patterns by ADAR2, ADAR1p110, ADAR1p150.**

A) In vitro transcribed RNA from *Rnf168, Knl,* and *Zkscan3* were refolded and *in vitro* edited with recombinant ADAR2, ADAR1p110, ADAR1p150. To determine A to I conversions, the RNA was reverse transcribed, overlapping fragments of the RNA were amplified using specific primers and PCR products were sequenced. G peaks were determined over A peaks. ADAR2 edits are highlighted in shades of green, ADAR1p110 edits in amber, and ADAR1p150 edits in lilac. Shadings indicate 25-50% editing (light), 50-75% editing (medium), and > 75% editing dark. On average ADAR2 edits more sites than either isoform of ADAR1. Only few editing events occur opposite unpaired cytosines (red dots). Red asterisks in the sequence highlight adenosines.

B) Sequence motif derived from the highly edited sites in the three RNAs. The motif follows the published nearest neighbor preferences previously described for ADARs.

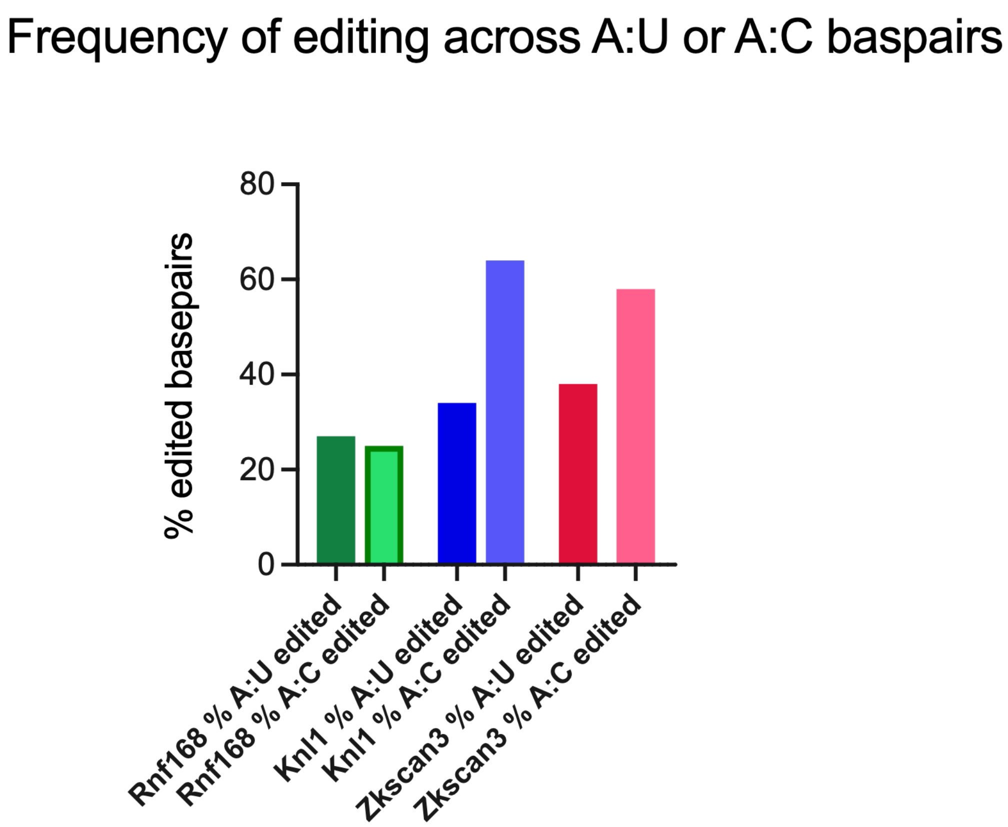

Supplementary figure 15: Occurrence of editing across A:U and A:C basepairs in %. The number of adenosines within longer double-stranded regions were counted and the percentage of adenosines at which editing occurred was compared for A:U basepairs and A:C basepairs. The percentage of edited A:U and A:C basepairs was roughly equal in Rnf168, while editing occurred at a higher frequency across A:C basepairs in Knl1 and Zkscan3.

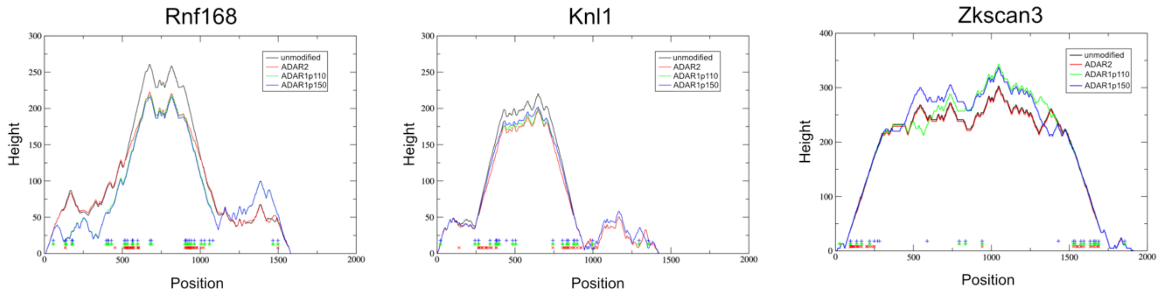

Supplementary Figure 16: Mountain plot of in silico folded RNAs with and without in vitro editing. Black line: folding of RNAs without inosines. RNAs with inosines introduced by ADAR2 (red line), ADAR1p110 (green line) or ADAR1p150 (blue line). The number of basepairs is indicated by the increase on the y-axes. As can be seen, the black (unedited) line is always leading to more basepairs than the edited (red, green, blue) RNAs.

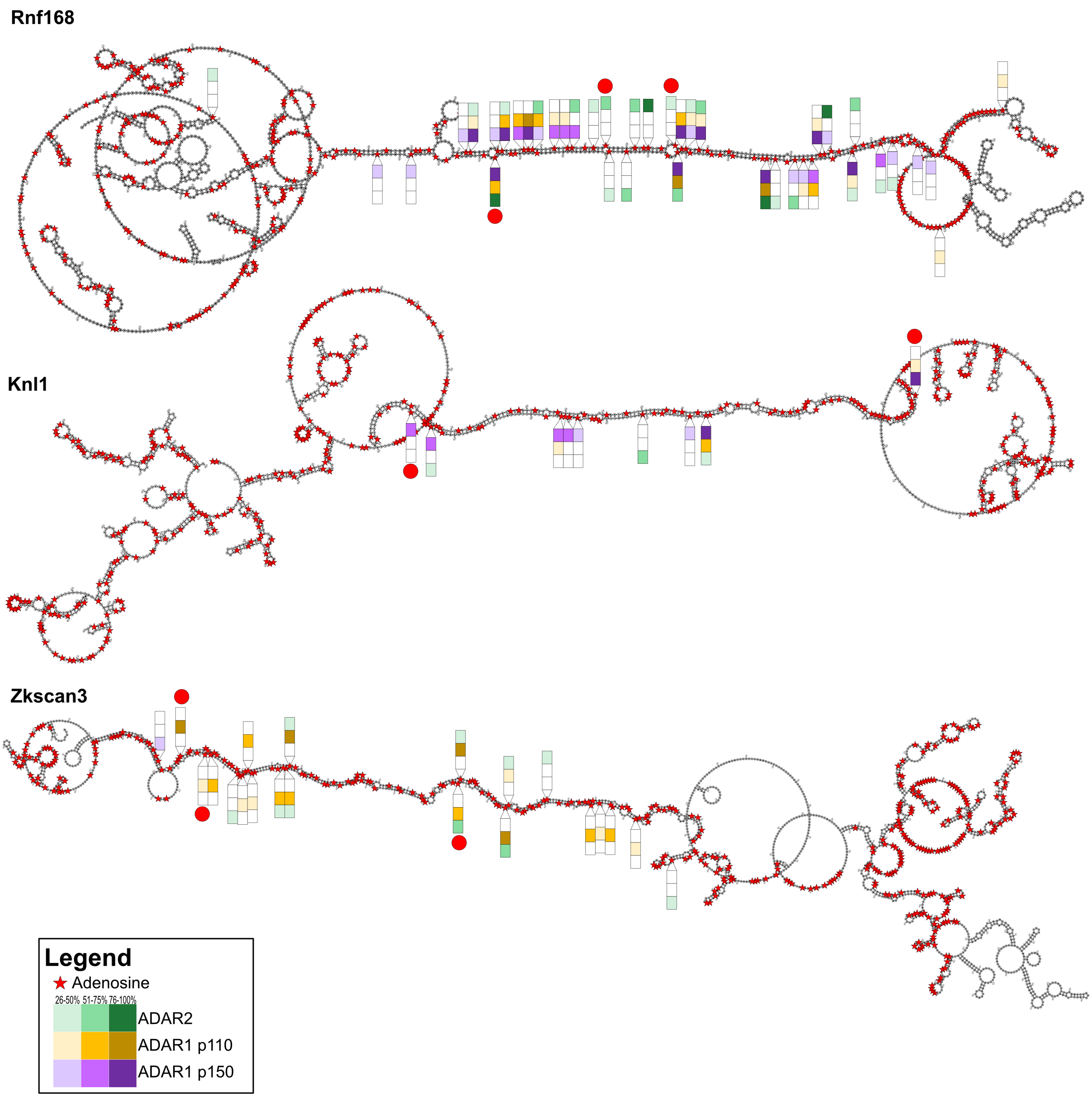

Supplementary Figure 17: *In cellulo* editing patterns by ADAR2, ADAR1p110, ADAR1p150. ADAR-less MEF cells were transfected or transduced with vectors expressing ADAR2, ADAR1p110 or ADAR1p150, respectively. 24 hrs post ADAR induction RNA was isolated, reverse transcribed and the 3' UTRs of endogenous *Rnf168, Knl1,* and *Zkscan3* sequences were amplified and sequenced. G peaks were determined over A peaks. ADAR2 edits are highlighted in shades of green, ADAR1p110 edits in amber, and ADAR1p150 edits in lilac. Shadings indicate 25-50% editing (light), 50-75% editing (medium), and > 75% editing dark. On average ADAR2 edits more sites than either isoform of ADAR1. Only few editing events occur opposite unpaired cytosines (red dots). Red asterisks in the sequence highlight adenosines.

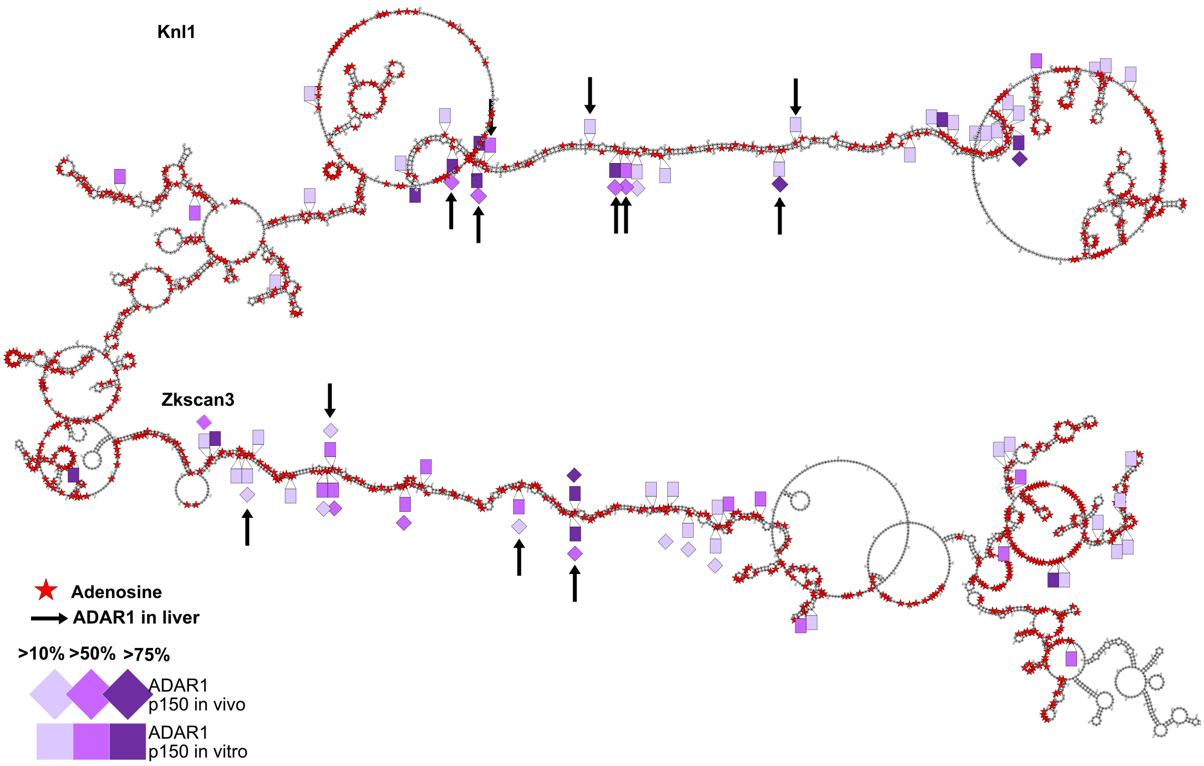

Supplementary figure 18: Comparison of editing patterns introduced by ADAR1p150 *in vitro,* in cells, and *in vivo*. In vitro transcribed RNA of *Knl1* and *Zkscan3* were edited with ADAR1p150 vitro. The same RNA fragments were amplified from cDNA generated from ADAR-less MEFs transduced with ADAR1p150 expressing lentivirus (rhomboids). Sites that are edited by ADAR1 in the liver of mice as previously reported and deposited in UCSC genome browser are highlighted by black arrows.

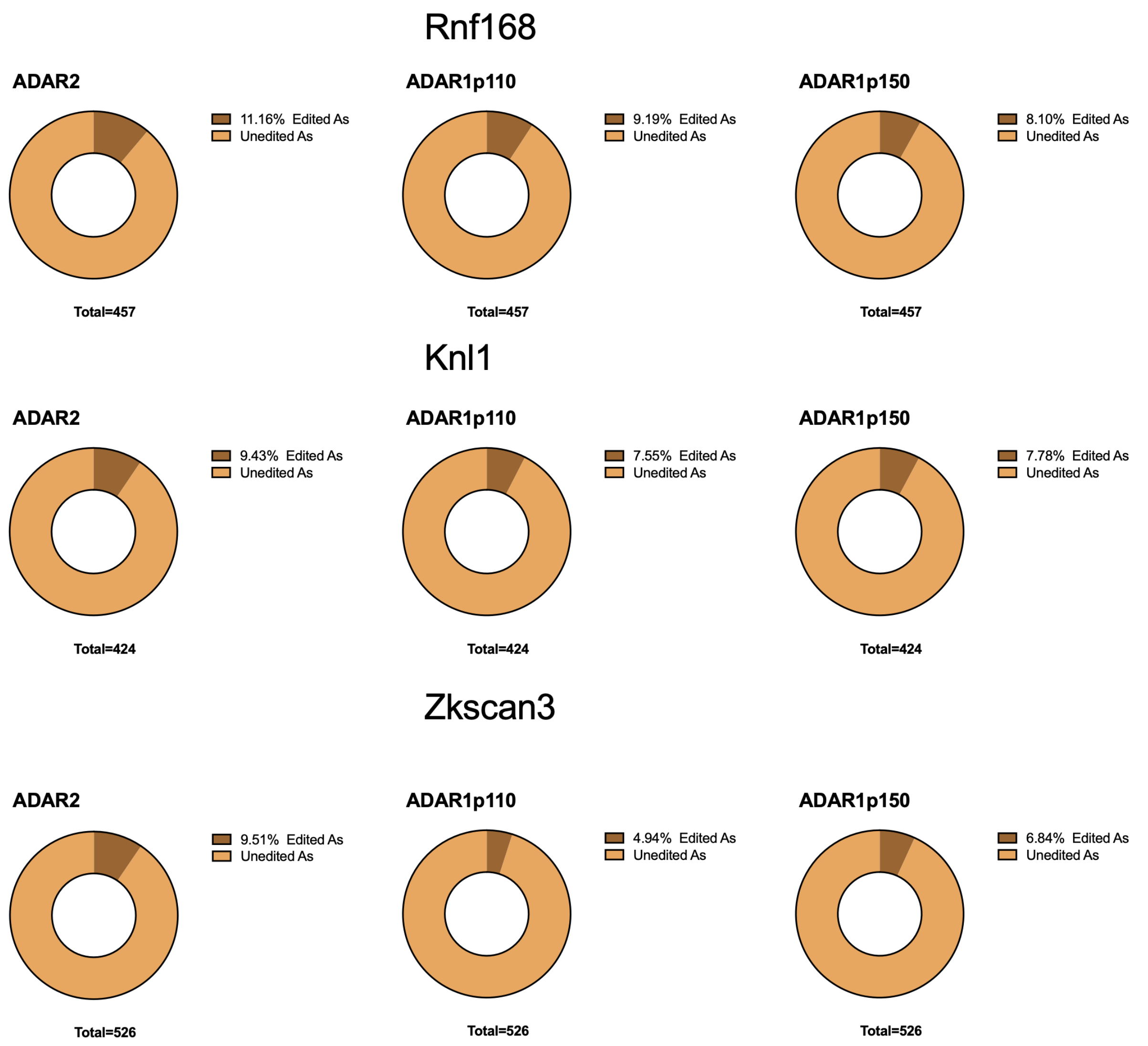

Supplementary Figure 19: Editing frequency by different ADARs *in vitro*. *In vitro* transcribed and *in vitro* edited RNAs were reverse transcribed and sequenced the number of adenosines in the sequences were counted and the percentage of positions at which editing was detected was counted for ADAR2, ADAR1p110 and ADAR1p150. Overall, the number of edits introduced by ADAR1p150 were lower than those introduced by ADAR2, indicating that 7-8% editing are sufficient to suppress Mda5 polymerization.

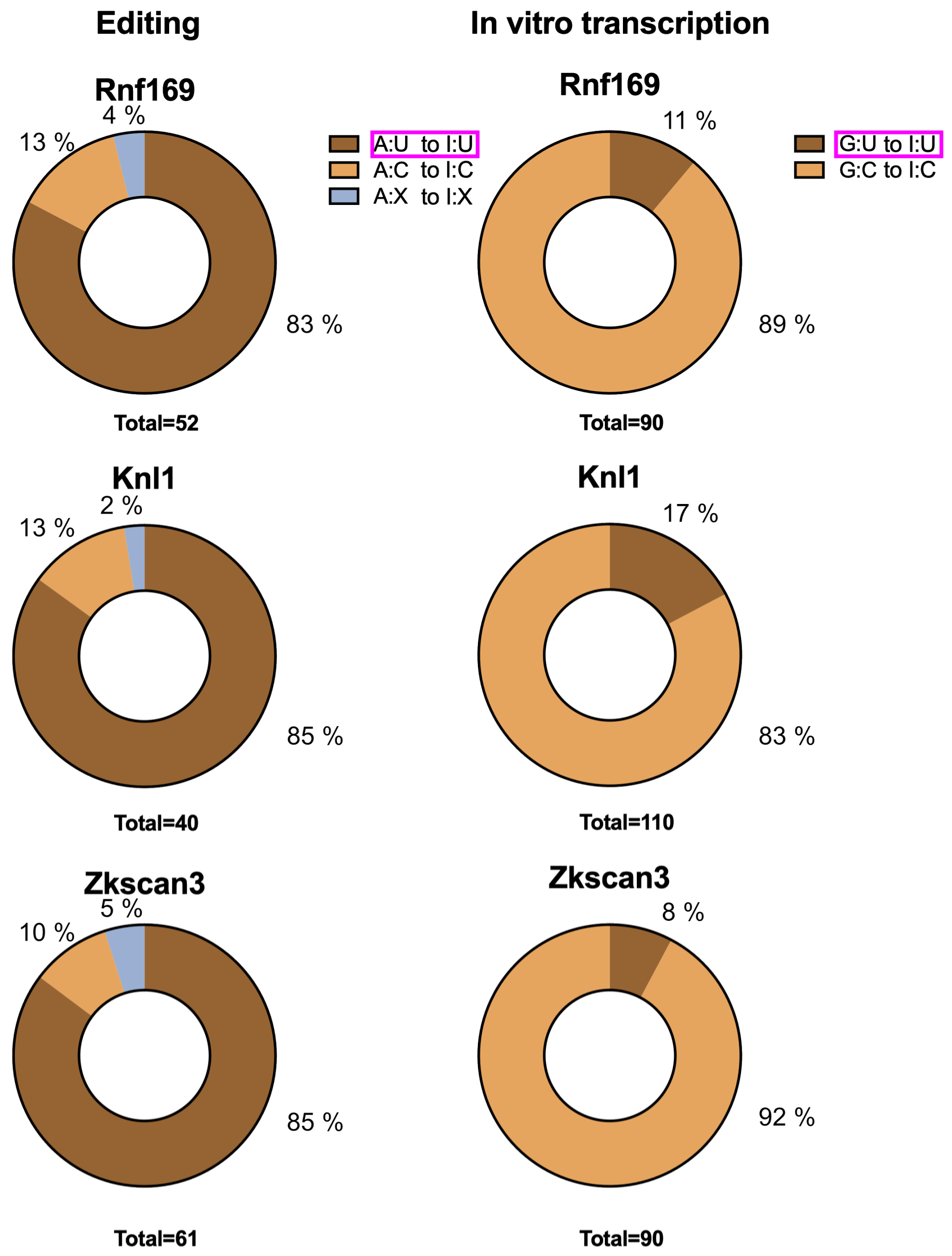

Supplementary Figure 20: Frequency of wobble I:U basepairs introduced by editing (left) and in vitro transcription (right).
The position of inosines was counted in the context of a folding prediction for the three edited RNAs RNF168, Knl1, and Zkscan3. On the right, the occurrence of guanosines that could be replaced by inosines during in vitro transcription and their putative basepair were counted. Editing mainly leads to the formation of I:U basepairs (brown, left), while in vitro transcription mainly leads to the formation of I:C basepairs (light brown, right).

1. Liang, Z., et al., *Generation of a new Adar1p150-/- mouse demonstrates isoform-specific roles in embryonic development and adult homeostasis.* RNA, 2023.
